# Genomic microdiversity of *Mycobacterium africanum* in Sénégal

**DOI:** 10.1371/journal.pntd.0014770

**Published:** 2026-10-26

**Authors:** Awa Ba Diallo, Joséphine Badiane, Mamadou Beye, Khardiata Gueye, Sébastien Cortaredona, Seynabou Lo, Michel Drancourt, Ghiles Grine, Makhtar Camara

**Affiliations:** 1 Mycobacteriology Unit, Bacteriology-Virology Laboratory, CHU Aristide Le Dantec Hospital, Dakar, Senegal; 2 Department of Biological and Pharmaceutical Sciences, Faculty of Medicine, Pharmacy and Odontology, Cheikh Anta Diop University, Dakar, Senegal; 3 MEPHI, Aix-Marseille University, Marseille, France; 4 Institut de Recherche en Santé, de Surveillance Épidémiologique et de Formations (IRESSEF), Dakar, Senegal; 5 Assistance Publique-Hôpitaux de Marseille, Marseille, France; 6 Aix-Marseille University, IRD, SSA, MINES, Marseille, France; 7 Aix-Marseille University, SSA, RITMES, Marseille, France; 8 IHU Méditerranée Infection, Marseille, France; 9 Faculty of Health Sciences, Gaston Berger University, Saint-Louis, Senegal; Institut Pasteur, FRANCE

## Abstract

**Introduction:**

*Mycobacterium africanum* is responsible for up to half of tuberculosis cases in West Africa and comprises four sub lineages. Its genomic diversity remains undescribed in Sénégal where *M. africanum* was first described and where the present study was conducted.

**Results:**

Whole Genome Sequencing (WGS) identified one *M. africanum* lineage L5 isolate and 50 *M. africanum* lineage L6 isolates, with a predominance of sub lineage L6.1.1 (24/50, 43.7%) across seven of eight regions of Sénégal. The Dakar region gathered all the sub lineages (L5.3, L6.1, L6.1.1, L6.1.3, L6.2.3, L6.3.1, L6.3.2, L6.3.3, unclassified L6), except for sub lineage L6.2. Further Single Nucleotide Polymorphisms (SNPs) analysis revealed substantial genetic diversity among the lineage L6 isolates with mean value of 668.54 (±239.61), while themedian was 465 SNPs (interquartile range = 400, median absolute deviation:163.09). According to the World health Organization Catalogue of mutations in the *Mycobacterium tuberculosis* complex and their association with drug resistance, WGS predicted antibiotic resistance in 16 strains. Comparing Drug Susceptibility Testing (DST) and WGS results showed complete concordance across 19 DST tested strains.

**Conclusion:**

This study highlights the diversity of *M. africanum* in Sénégal, with intra-lineage genomic variability and predicted antimicrobial resistance. The presence of synonymous mutations in the absence of resistance-associated variants suggests that WGS–based predictions of drug resistance should be interpreted with caution.

## Author summary

Senegal is the country where *Mycobacterium africanum* was first described. *Mycobacterium africanum* is a *Mycobacterium tuberculosis* complex pathogen responsible for a significant part of tuberculosis cases in some regions in West Africa, including Sénégal. Here whole genome sequencing of 51 *M. africanum* isolates recovered from eight regions in Sénégal, unprecedently found that one *M. africanum* lineage 5 isolate and a novel lineage 6 sub lineage, both participated to geographically structure the population of *M. africanum* in this country. This observation raises questions regarding weight of the multiple determinants of this situation, opening doors for next works aiming to question the zoonotic and environmental contributions.

## Introduction

*Mycobacterium africanum* (*M. africanum*) significantly contributes to the burden of tuberculosis in West Africa, where it accounts for up to half of all cases [1]. Initially described in Sénégal at the start of the 1960s [2], *M. africanum* is currently reported in 12 African countries where lineage 5 (L5, also reported as *M. africanum* West African 1), lineage 6 (L6, also reported as *M. africanum* West African 2) [3], lineage 9 (L9), and lineage 10 (L10) recently identified through in-depth phylogenetic analyses of isolates primarily isolated in the Democratic Republic of the Congo and in Belgium, are reported with distinct geographic distributions [4,5].

In Sénégal, previous spoligotyping technique studies have reported the predominant presence of *M. africanum* lineage L6 as observed in most neighbouring countries such as the Gambie [1,4,6]; but no whole genome sequence-based study has analysed these preliminary data in any depth [7,8]. This report provides such an analysis of *M. africanum* isolates from several regions in Sénégal and describes the fine genomic diversity of *M. africanum* in the country, including a report of one L5 isolate. It also compares phenotypic data with whole genome sequence-predicted resistance data, revealing a geographical structuration of the *M. africanum* population in Sénégal.

## Materials and methods

### Compliance with medical ethics regulations

The present study was conducted in accordance with the ethical principles of the Declaration of Helsinki [9]. No specific clinical samples were taken for this study, as all the clinical samples had been collected as part of the routine medical management of people suspected and confirmed to have tuberculosis. As this retrospective study was entirely anonymous and recorded no medical or biographical data, no external ethics advice was necessary for this study of mycobacteria isolates.

### Mycobacterial isolates and DST

*Mycobacterium tuberculosis* (*M. tuberculosis*) complex isolates, obtained from the mycobacteriology laboratory at the Aristide Le Dantec University teaching Hospital in Dakar, Sénégal, came from a series of 549 sputum samples which had been routinely collected between 2010 and 2015 from patients with pulmonary tuberculosis and those suspected of having pulmonary tuberculosis in eight regions of Sénégal (Dakar, Ziguinchor, Tambacounda, Diourbel, Saint-Louis, Kolda, Thiès, and Sédhiou). The sputum samples were cultured on Löwenstein-Jensen culture medium (Thermo Fisher Scientific, Breda, Netherlands) and incubated at 37 °C for up to two months. A preliminary identification was performed for positive cultures by fluorescent microscopic observation after auramine staining. The isolates confirmed under the microscope were then sub-cultured again on Löwenstein-Jensen culture medium to obtain a pure isolate, followed by confirmation of the *M. tuberculosis* complex using the rapid SD Bioline Ag MPT64 test (Abbott, USA) as previously described [10]. Identification of the relevant lineages was performed by spoligotyping using commercially available activated Biodyne C membranes with the 43 synthetic oligonucleotides covalently bound to the membranes (Ocimum Biosolutions, Huda Techno Enclave Madhapur, India) according to standardised methods, as previously described [11]. DNA extracted from 51 isolates previously identified as *M. africanum* using the cetyl trimethyl ammonium bromide (CTAB) method [12] was then stored at minus 20 °C for subsequent sequencing. Antimicrobial susceptibility testing was also performed for only 19 of these 51 isolates (due to technical constraints at that time) using the automated BACTEC Mycobacterial Growth Indicator Tube (MGIT) 960 system to evaluate their susceptibility to rifampicin (critical concentration: 1.0 µg/L), isoniazid (0.1 µg/L), streptomycin (1.0 µg/L), and ethambutol (5.0 µg/L), according to the manufacturer’s recommendations (Becton Dickinson Diagnostic Systems, Sparks, MD, USA), as previously described [13]. A laboratory quality control was carried out for each new batch of MGIT medium and enrichment incorporated standard strains of *M. tuberculosis* H37Rv, *Mycobacterium kansasii* and *Mycobacterium fortuitum*, according to the manufacturer’s instructions.

### WGS

DNA extracted as above from the *M. africanum* isolates, was mixed with TE buffer (10 mM Tris-HCl, 1 mM EDTA, pH 8.0), quantified using a NanoDrop 2000C Spectrophotometer (Thermo Fisher Scientific, Waltham, USA) and a 10 ng/µL aliquot in TE (Sigma-Aldrich) was stored at -20 °C until used. WGS was performed using the MiSeq version 4 and NovaSeq (Illumina, San Diego, California, USA) at the IHU Méditerranée Infection, Marseille, France. After storage, DNA was requantified using the Qubit dsDNA HS (high sensitivity) assay (Life Technologies, Carlsbad, California, USA) before libraries were prepared using Nextera XT kit following the manufacturer’s protocol (Illumina). DNA library concentrations were quantified as described above and normalised to 4 nM by adding Tris-HCl (10 nM, pH 8.5 with 0.1% Tween20). Thereafter, the indexed libraries of all 51 isolates were pooled into two groups to reach a final concentration of 12.5 pM and were loaded for sequencing onto the MiSeq sequencer for one group and on NovaSeq sequencer for the second group.

### Bioinformatics analysis

Direct quality control of the sequence reads was performed using FastQC [14] and Trimmomatic v0.39 for trimming and improving the quality of the reads [15]. Trimmed reads were analysed using TB-Profiler to perform alignment against the *Mycobacterium tuberculosis* H37Rv reference genome [16]. This analysis enabled lineage assignment, genome-wide variant detection, and prediction of antibiotic resistance profiles based on a curated mutation database [17,18]. The use of the --call_whole_genome option in TB-Profiler extended variant calling to the entire genome, allowing the detection of single nucleotide polymorphisms (SNPs) [16]. The tools bcftools (merge and filter) [19] were subsequently used to filter SNPs, retaining only majority variants, in order to consider only fixed SNPs and reduce false positives associated with sequencing errors or mixed populations [20]. In addition, SNPs located in genomic regions including PE/PPE gene families, repetitive regions, and other problematic loci described previously [21], were excluded from downstream analyses using a predefined mask of these regions. The resulting high-confidence SNP set was then used for pairwise comparisons between strains. Finally, SAMtools [22] and bcftools (consensus) [19] were used to generate consensus sequences for each sample. Manual inspection of SNP positions defining sub-lineages was performed using the IGV software (http://www.broadinstitute.org/igv/), to visually assess mapping quality, coverage at positions of interest, and the consistency of the observed variants.

DNA-DNA hybridisation (dDDH) values were determined using the Type Isolate Genome Server (TYGS) web tool [23], and a threshold of 70% dDDH was used for species classification [24,25]. In this study, the phylogenetic distribution of the genus *Mycobacterium* was inferred by aligning consensus sequences generated from SNP datasets in which previously identified problematic genomic regions had been masked [21], using Mugsy version 1.2.3 [26]. Phylogenetic analyses were performed using IQ-TREE2 version 2.2.0 with the Maximum Likelihood technique, implementing the General Time Reversible (GTR) model [27]. We used the bootstrap test (1,000 replicates) to determine the robustness of the branches, and the percentage of replicated trees where the connected taxa cluster was indicated next to the branches [28]. The obtained phylogenetic tree was represented using iTOL [29]. The interpretation of genetic variants associated with resistance to anti-tuberculosis drugs was performed using the reference catalogue of the World Health Organization for mutations in *M. tuberculosis* and their association with drug resistance. All the mutations not included in this catalogue were considered to not confer resistance to anti-tuberculosis drugs [30].

### Statistical analyses

Pairwise SNP distances were summarized using the median, interquartile range (IQR) and median absolute deviation (MAD), as the distribution of genomic distances was expected to be skewed and potentially influenced by extreme values. These measures were preferred to the mean and standard deviation because they are less sensitive to outliers and non-normal distributions, which are common in pairwise genetic distance data. This analysis was performed for all sites with at least four isolates to ensure a minimum level of statistical robustness for diversity estimation [31,32]. The potential cross-transmission of some isolates was assessed using the 12 SNPs threshold established by Walker et al., which allows discrimination between recent and more distant transmission events [33].

## Results

### Identification and typing of *M. africanum* isolates

A total of 51 *M. africanum* samples collected between 2010 and 2015 from patients residing in eight regions in Sénégal were investigated using WGS (Fig 1). Accurate identification of the isolates was confirmed by the results of spoligotyping and further WGS analysis, demonstrating that these 51 isolates had been successfully sequenced (S1 Table). Strain identification was accurately performed using TB-Profiler with the *M. africanum* genome ATCC 25420, a strain from Senegal (GenBank accession: GCF_002982335), for all the isolates. These 51 *M. africanum* isolates were further assigned to ten sub lineages including L5.3 (n=1, the sole isolate representative of lineage 5) and nine sub lineages in lineage L6 (n = 50), namely sub lineages L6.1.1 (n = 24; 43.7%), L6.1.3 (n = 7; 12.7%), unclassified L6 (n = 7; 12.7%), L6.3.1 (n = 5; 9.1%), L6.1 (n = 3; 5.5%), and one isolate in each of L6.2, L6.2.3, L6.3.2, and L6.3.3 (S2 Table). As for the unclassified lineage 6 and L6.1 isolates, phylogenetic analysis indicated a scattered distribution, with neither group forming a distinct cluster (Fig 2). SNP analysis revealed substantial genetic diversity between lineage 5 and lineage 6 isolates and among lineage 6 isolates. In the lineage 6 isolates, analysis of genetic variations showed an average of 561.45 SNPs per genome, with a median of 465 (MAD = 163.09) suggesting a slight asymmetry in the distribution. (S1 Fig and S3 Table). The raw data have been deposited in GenBank under the project accession number PRJNA1441484.

**Fig 1:**
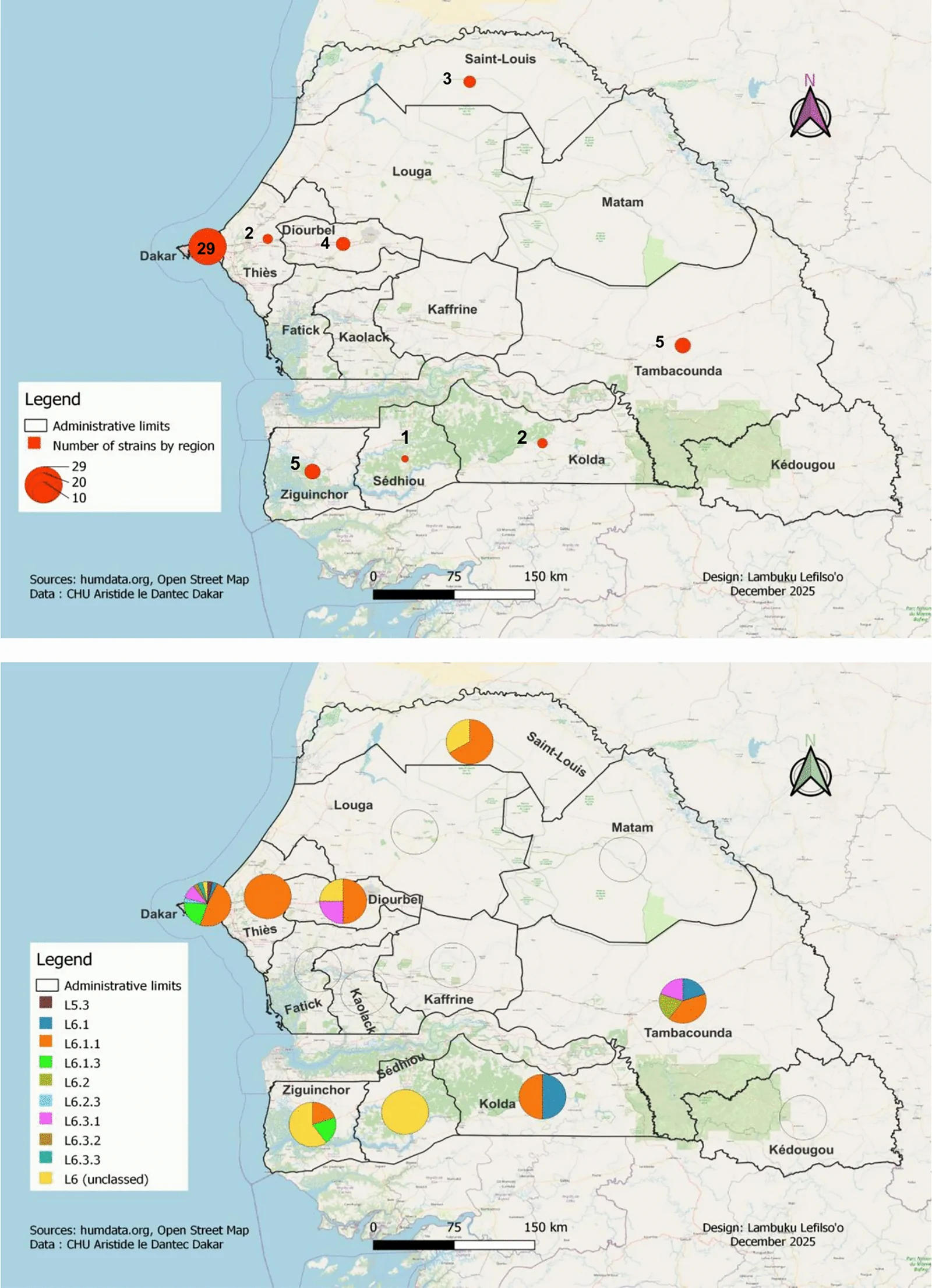
Geographical distribution of 51 *M. africanum* clinical isolates sampled in 2010-2015 in Sénégal at the sub lineage level. **A** illustrates the number of isolates collected in each of the studied regions, and **B** shows the distribution of sub lineages identified per region. Humdata (Senegal - Subnational Administrative Boundaries | Humanitarian Dataset | HDX) and QGIS (http://qgis.org) were used for the design of the maps and the visualization of the data.

**Fig 2:**
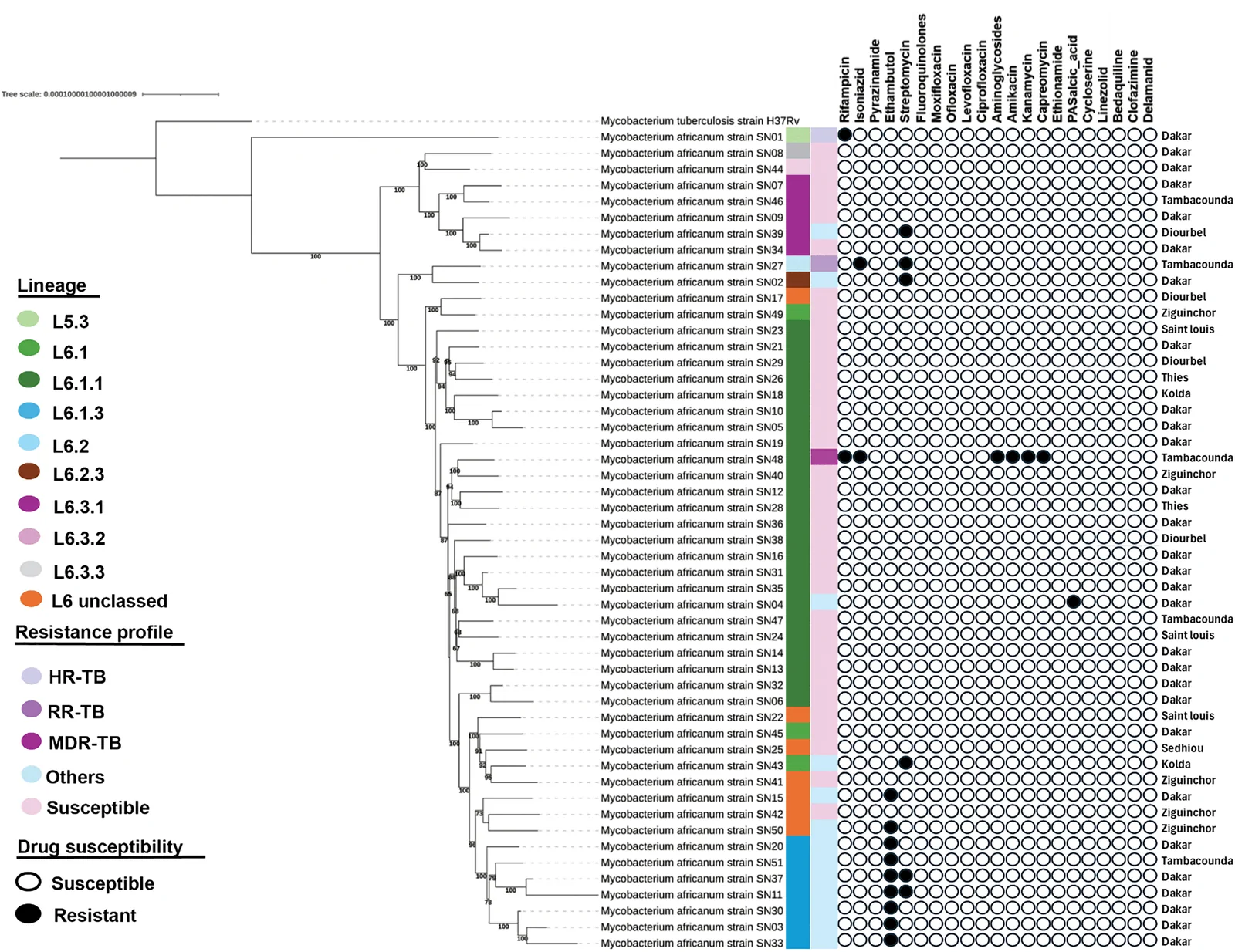
Phylogenetic tree and *in silico* prediction for antibiotic susceptibility pattern in 51 *Mycobacterium africanum* isolates sampled from 51 Senegalese tuberculosis patients in 2010-2015. Legend is reported to the left of the figure.

### WGS-based antibiotic susceptibility profiling

WGS predicted resistance to at least one antibiotic in 16/51 (31.4%) of the *M. africanum* isolates, including rifampicin monoresistance in one lineage L5.3 isolate, ethionamide-monoresistance in one L6.1.1 isolate, streptomycin monoresistance in three L6.2.3, L6.3.1, and L6.1 isolates, and ethambutol-monoresistance in seven isolates in unclassified L6 (two isolates) and L6.3.1 (five isolates) sub lineages. Two L6.3.1 isolates also exhibited resistance to ethambutol and streptomycin and one L6.2 isolate exhibited resistance to isoniazid and streptomycin. Finally, one isolate was predicted to be multidrug-resistant, exhibiting *in silico* resistance to rifampicin, isoniazid, amikacin, kanamycin, and capreomycin (Fig 2). Overall, the e*mbC*_p mutation Ala307Thr was the most frequent, affecting 56.3% of the resistant isolates and, notably, all seven of the L6.1.3 isolates and two unclassified L6 strains (S1 Table). According to the *Catalogue of mutations in the M. tuberculosis complex and their association with drug resistance*, WGS predicted antibiotic resistance in 16 strains; however, only four of them harboured mutations consistent with known resistance-associated variants. Specifically, one strain was classified as RR-TB (i.e., rifampicin-resistant TB) and exhibited mutations conferring resistance to rifampicin, aminoglycosides, and capreomycin; one strain was classified as HR-TB (i.e., rifampicin-susceptible, isoniazid-resistant TB) with mutations associated with isoniazid resistance; and two additional strains carried mutations associated to streptomycin resistance (S4 Table). In contrast, the remaining strains displayed only synonymous substitutions or mutations not currently associated with phenotypic resistance according to the catalogue. Accordingly, strain SN48, previously reported as multidrug-resistant, should be reclassified as an RR-TB strain based on the absence of additional resistance-conferring mutations.

Antibiotic susceptibility testing was limited to 19 isolates due to technical constraints. *In silico* predictions correlated with DST in 16 of the 19 tested isolates, including 15 susceptible isolates, while one isolate exhibiting streptomycin MIC > 1 µg/L (resistance) also exhibited a *rpsL*_p mutation Lys43Arg known to confer resistance to streptomycin and found in 26x equivalent sequencing depth and with 100% equivalent mutation frequency [34]. A high level of concordance was observed between phenotypic data and the interpretation of mutations based on the reference catalogue of the World Health Organization (S5 Table).

### Geography of sub lineages and resistance

The 51 *M. africanum* isolates were geographically distributed as follows: Dakar (n = 29; 56.4%), Ziguinchor (n = 5; 10.9%), Tambacounda (n = 5; 9.1%), Diourbel (n = 4; 7.3%), Saint-Louis (n = 3; 7.3%), Kolda (n = 2; 3.6%), Thiès (n = 2; 3.6%) and Sédhiou (n = 1; 1.8%). The district of Dakar city accounted for eight sublineages: L5.3 as the sole representative of lineage 5; L6.1.1 (n = 14), representing 45% of the isolates collected in this region; followed by L6.1.3 (n = 6), L6.3.1 (n = 3), L6.1 (n = 1), L6.3.2 (n = 1), L6.3.3 (n = 1), L6.2.3 (n = 1), and one unclassified L6 (S2 Table and Fig 1). In the Tambacounda region, there were five isolates sorted in four sub lineages, L6.1.1 (n = 2), L6.3.1 (n = 1), L6.1 (n = 1), and L6.2 (n = 1). In the Ziguinchor region, there were five isolates sorted in three sub lineages, L6.1.1 (n = 1), L6.1.3 (n = 1), and three isolates in unclassified lineage 6. In the Diourbel region, there were four isolates sorted in three sub lineages, L6.1.1 (n = 2), L6.3.1 (n = 1), and one unclassified L6 isolate. Manual exploration confirmed that all L6 sub lineage–defining SNPs were absent in these unclassified L6 isolates, despite adequate read coverage at the corresponding genomic positions, ruling out missing calls due to low sequencing depth (S3 Table). Only one isolate (SN17) showed a single read carrying the Rv2718 mutation (1/89 reads), which is insufficient to support sub lineage assignment and is consistent with sequencing noise rather than strain mixture.

In the Saint-Louis region, there were three *M. africanum* isolates sorted in two sub lineages, L6.1.1 (n = 2) and one unclassified L6 isolate. In the Thiès region, two L6.1.1 isolates were detected, in the Kolda region one L6.1 isolate and one L6.1.1 isolate was detected, and in the Sédhiou region, a single unclassified L6 isolate was identified (Fig 2). Further SNP analysis was performed only for the four regions in which four or more isolates had been investigated. A regional variability in genetic diversity was observed among the isolates. The Tambacounda region exhibited the highest median genetic distance (783), suggesting substantial divergence between strains, although with relatively low internal variability (MAD = 117.13). The Diourbel region was characterized by the greatest dispersion of genetic distances (IQR = 475.5; MAD = 343.22), indicating a highly heterogeneous bacterial population. Dakar showed an intermediate profile, with considerable genetic variability (IQR = 480; MAD = 217.94). In contrast, isolates from the Ziguinchor region displayed low genetic diversity, with a median of 441 and very limited dispersion (IQR = 25.2), consistent with a high level of genetic relatedness among strains (S3 Table). No region showed evidence of recent transmission dynamics of the identified *M. africanum* strains, with the minimum observed pairwise distance being 58 SNPs, well above the threshold previously established by Walker et al [31]. Accordingly, predicted drug-resistant isolates were detected in five regions (62.5%), namely Tambacounda (3/5, 60%), Kolda (1/2, 50%), Dakar (10/29, 34.5%), Diourbel (1/4, 25%), and Ziguinchor (1/5, 20%). No predicted resistant isolates were detected among the six isolates collected from Thiès, Saint-Louis, and Sédhiou. In Dakar, the L6.1.3 sub lineage (6/10; 60%) accounted for most resistant isolates, followed by L5.3 (1/10, 10%), unclassified L6 (1/10, 10%), L6.1.1 (1/10, 10%), and L6.2.3 (1/10, 10%). In Tambacounda, three predicted resistant isolates belonged to sub lineages L6.2, L6.1.1, and L6.1.3 whereas in Diourbel, Kolda, and Ziguinchor, predicted resistant isolates belonged to sub lineages L6.3.1, L6.1 (1/2), and unclassified L6, respectively. The fifteen isolates predicted to be resistant to ethambutol or streptomycin, had been collected from Dakar (n = 10), Tambacounda (n = 02), Kolda (n = 01), and Ziguinchor (n = 01). It should be noted that 50% of the variants identified by WGS corresponded to synonymous mutations, not directly associated with a resistance phenotype, but potentially warranting continuous molecular surveillance.

## Discussion

*M. africanum* was first describted in Sénégal after its phenotypic distinction from both *M. tuberculosis stricto sensu* and *Mycobacterium bovis* [2]. This study, incorporating WGS analyses, confirmed the prevalence of *M. africanum* in its country of origin with a notable, previously reported predominance of sub lineage L6 (more precisely of sub lineage L6.1.1), here representing 98% of analysed isolates. The different sub lineages here reported in Sénégal fall within the genomic diversity of *M. africanum* previously reported in West Africa [3]. In addition, the study also added *M. africanum* lineage 5 and a novel sub lineage 6 to the repertoire of *M. africanum* lineages and sub lineages circulating in Sénégal, indicating that the genomic landscape of *M. africanum* is not yet fully known in the country. It is notable that the novel sub lineage L6 was detected in three regions bordering rivers, suggesting that there may be environmental or ecological characteristics associated with this novel sub lineage beyond that of specific adaptations to the local populations [1,35].

Pioneering studies have, indeed, reported a geographical structuration of *M. africanum*. One large study indicated that phenotypically defined *M. africanum* isolates were more frequently isolated from tuberculous patients living along the Sénégal River (prevalence 46.52%) than in the Thiès region (prevalence 24.18%), the Dakar region (prevalence 23.23%), the Sine Saloum region (prevalence 18.52%) or the Diourbel region (10.35%) [36]. Our study did not cover the Sénégal River region (with only three *M. africanum* isolates from Saint-Louis) yet also found regional differences in the genomic diversity of *M. africanum*, including sub lineage diversity and differences in predicted antibiotic resistance. While the Dakar region exhibited the largest *M. africanum* genomic diversity marked by sub lineage diversity, this situation may be biased by the attractive effect of the capital of Senegal, which provided the majority of *M. africanum* investigated here.

By contrast, the situation was unbiased in other regions of Sénégal, although results limited by relatively small sample size of studies isolates. The Tambacounda region exhibited the highest diversity as measured by SNPs. The Tambacounda region is a large region (42 364 km^2^, 98 8193 habitants) characterised by contrasted ecosystems from tropical forests to semi-arid landscapes and is an historical crossroads for populations of different ethnic backgrounds. First-line WGS analyses of five *M. africanum* isolates collected in this region disclosed four different sub lineages of lineage L6, showing high variability despite the small number of strains collected in this region. Most of the synonymous mutations identified through WGS analysis were in the *embC* gene, and these were observed in the majority of strains collected in the Tambacounda region. In this region, the flora comprises tropical plants which, when fermentation by bacteria such as *Clostridium acetobutylicum,* results in the biosynthesis of butanol, the natural homologue to ethambutol which is a semi-synthetic antibiotic. This observation suggests that in some ecosystems of the Tambacounda region, predicted butanol-resistant *M. africanum* isolates can be found in patients undergoing polychemotherapy, including treatment with ethambutol, as recommended [37]. Although one study conducted in Sénégal failed to find *M. africanum* in animals or in the environment [36], another has reported the presence of *M. africanum* in chimpanzees and guenons in West Africa [38]. This leaves open the possibility of environmental reservoirs for *M. africanum*. SNP-based analyses reveal a high level of genetic heterogeneity among *M. africanum* strains circulating in Senegal, consistently observed across all studied regions. All isolates exhibit pairwise genetic distances well above the threshold defined by Walker et al. [33] for inferring recent transmission. This suggests transmission dynamics characterized by diverse circulating lineages rather than recent transmission events. The Tambacounda region which showed the highest diversity is also a crossroad for populations and the local genetic background and complex interplays with different environments must play a role in the genomic diversity of *M. africanum*. Different intestinal microbiota has been found in patients infected with *M. tuberculosis* and *M. africanum* in Mali, where *M. africanum*-infected patients exhibited a decreased diversity and an increase in Enterobacteriaceae compared to a healthy control group of ten individuals [39]. We previously reported that *Enterococcus mundtii* was exclusively found in individuals without tuberculosis in Burkina Faso, linked to the secretion of bacteriocins active against *M. tuberculosis* complex mycobacteria [40,41].

In this study devoid of sampling bias, the systematic collection of *M. africanum* isolates allowed to estimate a 9% prevalence of *M. africanum* among the *M. tuberculosis* complex. This estimation is quite different from the one made by Diop *et al*., reporting a 21% prevalence for *M. africanum* in 1976 [35]. A further 2024 study indicates an even more marked decrease, with an estimated 7.4% prevalence (J. Badiane and collaborators, personal communication). This trend in decreasing prevalence of *M. africanum* among the *M. tuberculosis* complex in Sénégal has also been observed in different countries such as neighbouring Mali [42]. Whether this trend is artificial, due to a diagnostic underestimation, possibly due to the phenotypic particularities of *M. africanum;* or a real one suggesting the progressive replacement of *M. africanum*, by other lineages of *M. tuberculosis*, remains to be studied [43].

Our findings indicate that the rare detection of *Mycobacterium africanum* lineage L5 in Senegal is broadly consistent with the distribution patterns reported in neighbouring West African countries [1,4], including the Gambia, which is geographically enclosed within Senegal, providing a particularly relevant epidemiological context for interpreting the distribution of *M. africanum* lineages in the region. This apparent regional pattern highlights the need for further genomic and epidemiological investigations to better characterize the distribution, population structure, transmission dynamics, and ecological determinants of *M. africanum* across West Africa. In this context, an integrated “One Health” approach to investigating the natural dynamics of *M. africanum* in Sénégal and West Africa is warranted, with the two-fold view of improving surveillance and prevention of this neglected pathogen in this region of the world.

## Supporting information

S1 Fig Heatmap of pairwise SNP distances with hierarchical clustering of 51 *M. africanum* isolates sampled from tuberculosis patients in Sénégal in 2010–2015. (TIF)

S1 Table Whole genome sequencing based antibiotic susceptibility profiling of 51 *M. africanum* isolates in Sénégal between 2010 and 2015. (XLSX)

S2 Table Distribution of *M. africanum* isolates in eight regions of Sénégal. This table shows the identified lineages and sub lineages, along with the number and proportion (n/total number of collected strains) of each lineage and sub lineage.
(XLSX)

S3 Table SNP matrix of 51 *M. africanum* isolates in Senegal in 2010–2015. (XLS)

S4 Table Coverage and predicted mutation frequency of sixteen *Mycobacterium africanum* resistant isolates in this study. (XLSX)

S5 Table Antibiotc susceptibility profiling. Whole genome vs DST on nineteen *Mycobacterium africanum* isolates tested with the BACTEC MGIT 960 system.
(XLSX)
