# Supplementary material for: Genomic microdiversity of Mycobacterium africanum in Sénégal
Source: PLoS Negl Trop Dis. 2026 Oct 26;20(10):e0014770. doi: 10.1371/journal.pntd.0014770 (PMC13727044; doi:10.1371/journal.pntd.0014770)
Supplement: Supplementary file 1 [file pntd.0014770.s008.docx]

Dear Editor,

Please find enclosed the revsied version of PNTD-D-25-01817 entitled « 
Genomic microdiversity of *Mycobacterium africanum* in Sénégal. » along with the answers of the authors to the Editor and Reviewers’comments.

Please include the following items when submitting your revised manuscript:

* A rebuttal letter that responds to each point raised by the editor and reviewer(s). You should upload this letter as a separate file labeled 'Response to Reviewers'. This file does not need to include responses to any formatting updates and technical items listed in the 'Journal Requirements' section below.

**Authors’answer : The present rebuttal letter is enclosed in the submission.**

* A marked-up copy of your manuscript that highlights changes made to the original version. You should upload this as a separate file labeled 'Revised Manuscript with Track Changes'.

**Authors’answer : Done.**

* An unmarked version of your revised paper without tracked changes. You should upload this as a separate file labeled 'Manuscript'.

**Authors’answer : Done.**

**Reviewers' Comments:**

Reviewer #1: 1. Are the objectives of the study clearly articulated with a clear testable hypothesis stated?
Yes. The objective—to describe the genomic diversity of Mycobacterium africanum in Senegal using WGS and compare phenotypic and genotypic resistance—is clearly stated in the Introduction. While the hypothesis is implicit rather than explicitly formulated, the aim is testable.
2. Is the study design appropriate to address the stated objectives?
Yes. A retrospective design using isolates collected during routine surveillance is appropriate for assessing genomic diversity and resistance patterns.
3. Is the population clearly described and appropriate for the hypothesis being tested?
Partially. The manuscript specifies that isolates were obtained from eight regions in Senegal between 2010 and 2015, but it does not detail the type of clinical specimens or patient characteristics. This limits epidemiological context.
4. Is the sample size sufficient to ensure adequate power to address the hypothesis being tested?
For a descriptive genomic study, 51 isolates provide meaningful insights into diversity, though the authors acknowledge limitations for regional comparisons. For statistical comparisons of resistance rates, the sample size is modest but acceptable for exploratory analysis.
5. Were correct statistical analyses used to support conclusions?
Yes. The manuscript uses binomial proportion tests and McNemar’s test with Benjamini–Hochberg correction for multiple comparisons, which are appropriate for the data presented.
6. Are there concerns about ethical or regulatory requirements being met?
No major concerns. The study uses anonymized retrospective isolates collected during routine care, and the authors state compliance with the Declaration of Helsinki. No patient identifiers or sensitive data were included.

**Authors’answer : The authors acknowledge these positive comments, including the one regarding statistical analyses.**

Reviewer #2: objectives clear, sample size ok
inappropriate use of assemblies, poor statistical description of data

**Authors’answer : Following this comment, authors improved the clarity and transparency of the statistical description, and revised all relevant tables to explicitly report absolute numbers, frequencies, and percentages of isolates. This allows readers to clearly identify the denominators used and to better interpret the reported proportions and statistical comparisons. The Results section and tables have been updated accordingly (Lines 245-246 ; Tables S6 & S7).**

Reviewer #1: 1. Does the analysis presented match the analysis plan?
Yes. The results follow the described methods: WGS for lineage and SNP analysis, DST for 19 isolates, and statistical comparisons of resistance rates.
2. Are the results clearly and completely presented?
Yes. The main findings are well described.
3. Are the figures (Tables, Images) of sufficient quality for clarity?
Partially. Figures 1 and 2 are informative but could benefit from additional details (see general comments). Figure 3 is difficult to read and should be improved for resolution and clarity.

**Authors’answer : The authors amended Figures 1 & 2 according to comments below ; Figure 3 has been deleted.**

Reviewer #2: results incomplete (info on coverage for all samples)
possibly manually modified figure (Fig. 2)

**Authors’answer : The authors took into consideration these remarks to improve Figure 2 which is not a modification: it was simply created manually from an Excel file.**

**Conclusions**

Reviewer #1: 1. Are the conclusions supported by the data presented?
Yes. The conclusions about genomic diversity, lineage distribution, and emerging resistance are consistent with the WGS and DST data presented.

**Authors’answer : The authors acknowledge this positive comment.**

2. Are the limitations of analysis clearly described?
Partially. The discussion acknowledges geographic bias (Dakar overrepresented) and small sample size for some regions, but does not explicitly address the lack of temporal data or clinical specimen details.

**Authors’answer : These isolates have been collected as part of routine diagnostic procedures, therefore requiring no informed consent that would allow providing more details on the clinical samples. That said, authors now clarify that samples were sputum samples (line 101, line 104) collected from patients confirmed or suspected with pulmonary tuberculosis (line 102) (“Mycobacterial isolates and DST” section).**

3. Do the authors discuss how these data can be helpful to advance our understanding of the topic under study?
Yes. The authors emphasize the importance of genomic surveillance and suggest a “One Health” approach for future research, which advances understanding of M. africanum epidemiology.

**Authors’answer : The authors acknowledge this positive comment.**
4. Is public health relevance addressed?
Yes. The manuscript highlights implications for tuberculosis control in Senegal and West Africa, particularly regarding drug resistance prediction and surveillance.

**Authors’answer : The authors acknowledge this positive comment.**

Reviewer #2: Global conclusions such as L6 prevalence as compared to L5 supported.
discssion interesting regarding ecology of the bacteria
public Health relevant

**Authors’answer : The authors acknowledge this positive comment.**

**Editorial and Data Presentation Modifications?**


Reviewer #1: Several points could be clarified or improved:

1. Clarification of isolate selection: The manuscript states that 51 isolates were analyzed from a pool of 549 samples collected between 2010 and 2015. It is unclear whether these 51 isolates represent all M. africanum isolates identified during this period or a subset selected based on specific criteria. Please clarify the selection strategy and whether the sampling was exhaustive or biased.

**Authors’answer : The reviewer is perfectly right. To the best of authors ‘knowledge, there was no sampling biases (line 353). Also, the authors clarify that there was no selection of isolates spoligotyped as *M. africanum* in the collection period thus representing all such *M. africanum* isolates in the collection period (Line 353). Therefore, data here reported allowed for an estimation of the prevalence of *M. africanum* in the *M. tuberculosis* complex in Sénégal in the collection period (line 354). Out of the 549 isolates collected during this period, spoligotyping classified 55 isolates as *Mycobacterium africanum*; of which 51 were confirmed by WGS whereas three could not be identified due to insufficient genetic material, and one was considered representative of a novel *Mycobacterium* species unreported in this paper due to the unavailability of the isolate. These points were clarified in the "Mycobacterial isolates and DST" paragraph, line 116.**

2. Source of clinical samples and temporal distribution: The manuscript does not specify the type of clinical specimens from which the isolates were obtained. This information is important for understanding the epidemiological context. Additionally, providing the temporal distribution of isolates across centers would allow readers to relate the evolution of mutations (SNPs) to time and geography.

**Authors’answer : The reviewer is perfectly right and these precisions are now given in the revised version (Lines 106 and 108).**

3. Justification for AST on only 19 isolates: AST was performed on 19 out of 51 isolates, but no rationale is provided for this limited testing. Please explain why AST was not performed on all isolates.

**Authors’answer : Antibiotic susceptibility tests were redone using the automated BACTEC Mycobacterial Growth Indicator Tube, limited by reagent shortages, clarified in lines 227-228.**

4. Presentation of AST Results: Detailed AST results are currently relegated to supplementary tables. Given their importance for clinical and epidemiological interpretation, a summary table comparing phenotypic AST and WGS predictions should be included in the main manuscript.

**Authors’answer : The reviewer is perfectly right, a novel Table 1 in now included in the revised version of the manuscript.**

5. Figure 1: Figure 1 shows sublineage distribution but does not indicate the number of isolates per region. Adding these counts would improve clarity and allow readers to better interpret the geographic structuring.

**Authors’answer : The reviewer is perfectly right and these precisions are now given in the novel version of Figure 1.**

6. Figure 3:The historical distribution map is interesting but difficult to read. Consider improving resolution and enhancing the legend for better interpretability.

**Authors’answer : Figure 3 has been deleted from the revised version of the manuscript.**

Reviewer #2: (No Response)

**Summary and General Comments**

Reviewer #1: This manuscript by Ba Dialo et al. addresses an important and underexplored topic: the genomic diversity of Mycobacterium africanum in Senegal, its country of origin. The study is well-conducted, combining whole genome sequencing (WGS) and phenotypic antimicrobial susceptibility testing (AST) to provide novel insights into lineage distribution and antimicrobial resistance. The work is original, as no previous WGS-based study has characterized M. africanum isolates from Senegal. The findings have significant implications for tuberculosis surveillance and control in West Africa.

**Authors’answer : The authors ackowledge this general positive comment.**

Reviewer #2: The article by Diallo et al describes the genomic diversity of Mycobacterium africanum in Senegal using Whole genome sequencing data. This type of study is important as Mtb diversity in this country has been mainly described using genetic footprints from CRISPR region (spoligotyping). The study uses Illumina sequencing on 51 DNA extracts. It confirms that L6 is the major lineage belonging to M. africanum species, but documents for the first time the presence of rare L5 strains (n=1 in Senegal). It also confirms the presence of several L6 sublineages circulating in the country. Antibiotic resistance profiles are derived from TB-Profiler analysis. The distribution of M. africanum diversity is commented in face of the ecological context of strains, proposing hypotheses to account for intriguingly high resistance level to ethambutol. The article is globally sound and well written. However, many imprecisions weaken the reliability of the conclusions.

**Authors’answer : The authors acknowledge this general positive comment, and will do their best to adress the reviewers’ comments and clarify any imprecision in the initial version.**

Major comments:
-the information derived from each bioinformatical analyses is unclear. The way that lineages and antibiotic resistance are informed resembles TB-Profiler outputs but the authors suggest that it derives from the genome assemblies they constructed. TB-Profiler is meant to process directly short-reads sequencing data and assemblies derived from short reads. So, a good practice would be to use TB-Profiler to interpret lineage and antibiotic resistance. So better explanations on what has been done is needed, and rigorous choices on methodology are required. In addition, statistical data retrieved from TB-Profiler need to be presented extensively. A key information is the depth of sequencing. This information is needed for all strains and not only resistant ones. In addition, when mean depth (referred to as mean coverage) is low, a manual exploration of the alignment is crucial to confirm or reject SNP presence. This should be done

**Authors’answer : The reviewer is perfectly right, and these technical points have now been clarified in the Materials and Methods section of the revised manuscript (Lines 146–159). As indicated, drug resistance and lineage analyses were performed using TB-Profiler, and this is now explicitly stated to improve clarity. In addition, following the reviewer’s suggestion, the complete TB-Profiler results for all samples have been included as supplementary material (Table S5).**

-assemblies could help exploring the quality of the data and other features such as the presence of specific regions present in L5 or L6 but absent from other lineages. Yet authors should be cautious about the quality of their assemblies as they harbour coverages (the total length of contigs as compared to H37Rv length) well above the expectation (expectation is less than 110%). It is likely that in the current assemblies, several sequences are present in multiple copies where only one exists in the genomes. In turn, the samples could be contaminated, which could also lead to false variant inferences. Hence a more extensive presentation of the outputs and/or a more thorough use of these results are needed.

**Authors’answer : We thank the reviewer for this comment. The reference genome cited (*Mycobacterium tuberculosis* H37Rv) belongs to lineage L4 and is therefore not directly comparable to the genomes analyzed here, which belong to *Mycobacterium africanum* lineages L5/L6. These lineages harbour lineage-specific genomic regions absent from H37Rv, which can partly explain the larger assembly sizes observed. To address the possibility of contamination or sequence duplication, we performed a systematic quality assessment using CheckM (Table S6). All assemblies showed high completeness and very low contamination (<1%), with no evidence of significant gene duplication or strain heterogeneity, arguing against contamination or major assembly artefacts (Lines 183 –186). Finally, variant calling and drug resistance prediction were performed using read-based analyses (TB-Profiler) rather than de novo assemblies, thereby minimizing this risk.**
-Fig. 2 tree has a hidden part, and visible shapes seem incompatible. It looks like a manually modified figures which casts doubt on its scientific reliability…

**Authors’answer : The authors confirm there is no hidden part in this Figure which is by no way any modification of an underlying (hidden) figure, having been manually created strating from an Excel file.**

-additional insight should be provided as per how much the gathered data conform, modify or refine L6 history of diversification proposed by Coscolla et al. Of note, in that study, Senegal is not presented as the origin of L6. This point should be amended and/or better discussed.

**Authors’answer :** **The reviewer is perfectly right, this point is now discussed in the Discussion section (Lines 289-291). Although the article is not available on PubMed, authors such as de Jong support that the first African-type mycobacterial strain was isolated in Sénégal by Castet et al. in 1968.**

-the authors made the choice of declaring a strain resistant as soon as some reads carry a resistance mutation. It is important to consider and discuss in the article whether some little contamination with another strain could account for these rare reads carrying the resistance.

**Authors’answer : All genomes were pre-assessed with CheckM, showing very low contamination (<1%) and no strain heterogeneity, making contamination unlikely. In addition, TB-Profiler reports resistance mutations only when supported by sufficient read depth and allele frequency (typically ≥10%), minimizing the risk that rare reads from minor contamination lead to false resistance calls.**

Additional comments:
-some information describing the relative prevalence of M. africanum as compared to other lineages would increase the interest of the study

**Authors’answer : This has been included in the discussion (lines 340–348)**
-S1 figure. Clarify the identity of the outgroup.

**Authors’answer : There was no outgroup.**

-Nature of L6 unclassified should be discussed: when they group with a strain of an identified sublineage on the phylogenetic reconstruction, with a branch with strong bootstrap support, it is parsimonious to infer that they belong to the same sublineage. Their not carrying the classifying SNP can be due either to the corresponding SNP not been identified because of low depth at the corresponding position, or to the strain being a mixture of several strains from distinct sublineages so that no sublineage SNP has a sufficient prevalence to call the corresponding classifying SNP, or to the SNP(s) used for classification being inaccurate, allowing only to classify part of the population and/or misclassifying some strains carrying the SNP associated to the sublineage but due to an independent mutation (homoplasy). To make this clear, manual exploration of the alignments of each unclassified sample at classifying SNP positions is required.

**Authors’answer : Sublineage classification in *Mycobacterium africanum* L6 is based on the presence of specific signature SNPs: L6.1 (Rv1332, Rv1762c, Rv3003c), L6.2 (Rv2718, Rv2042), and L6.3 (Rv1811, Rv2629). We manually inspected all these classifying SNP positions in all L6 unclassified strains. All signature SNPs were absent in these isolates, despite adequate read coverage at the corresponding genomic positions, ruling out missing calls due to low depth. Only one isolate (SN17) showed a single read carrying the Rv2718 mutation (1/89 reads), which is insufficient to support lineage assignment and is consistent with sequencing noise rather than strain mixture. Importantly, CheckM analyses showed no evidence of strain heterogeneity or contamination across all genomes. In contrast, phylogenetic reconstruction relies on whole-genome variation and reflects overall evolutionary relatedness. Therefore, discrepancies between phylogenetic clustering and lineage assignment based on a small set of signature SNPs may arise. This point has now been clarified in the revised manuscript (Lines 264-271).**

PLOS authors have the option to publish the peer review history of their article ([what does this mean?](https://track.editorialmanager.com/CL0/https:%2F%2Fjournals.plos.org%2Fplosntds%2Fs%2Feditorial-and-peer-review-process%23loc-peer-review-history/1/010f019ae58337e3-224267fc-0e75-49db-a032-d5ecf795a32b-000000/-Tj9cmqi_m_VLQ7OT4r7S0XnUhsTvSkrO3j_FcGfYzs=240)). If published, this will include your full peer review and any attached files.

**Figure resubmission:**

While revising your submission, we strongly recommend that you use PLOS’s NAAS tool (https://ngplosjournals.pagemajik.ai/artanalysis) to test your figure files. NAAS can convert your figure files to the TIFF file type and meet basic requirements (such as print size, resolution), or provide you with a report on issues that do not meet our requirements and that NAAS cannot fix.

After uploading your figures to PLOS’s NAAS tool - https://ngplosjournals.pagemajik.ai/artanalysis, NAAS will process the files provided and display the results in the "Uploaded Files" section of the page as the processing is complete. If the uploaded figures meet our requirements (or NAAS is able to fix the files to meet our requirements), the figure will be marked as "fixed" above. If NAAS is unable to fix the files, a red "failed" label will appear above. When NAAS has confirmed that the figure files meet our requirements, please download the file via the download option, and include these NAAS processed figure files when submitting your revised manuscript.

**Reproducibility:**

To enhance the reproducibility of your results, we recommend that authors of applicable studies deposit laboratory protocols in protocols.io, where a protocol can be assigned its own identifier (DOI) such that it can be cited independently in the future. Additionally, PLOS ONE offers an option to publish peer-reviewed clinical study protocols. Read more information on sharing protocols at <https://plos.org/protocols?utm_medium=editorial-email&utm_source=authorletters&utm_campaign=protocols>

**Authors’answer : The authors described their methods in the appropriate section allowing for reproducibility; including amendments following reviewers’ recommendations.**

**Journal Requirements:**

1) Please upload all main figures as separate Figure files in .tif or .eps format. For more information about how to convert and format your figure files please see our guidelines: 
https://journals.plos.org/plosntds/s/figures

**Authors’answer : Done.**

2) Some material included in your submission may be copyrighted. According to PLOSu2019s copyright policy, authors who use figures or other material (e.g., graphics, clipart, maps) from another author or copyright holder must demonstrate or obtain permission to publish this material under the Creative Commons Attribution 4.0 International (CC BY 4.0) License used by PLOS journals. Please closely review the details of PLOSu2019s copyright requirements here: PLOS Licenses and Copyright. If you need to request permissions from a copyright holder, you may use PLOS's Copyright Content Permission form.
Please respond directly to this email and provide any known details concerning your material's license terms and permissions required for reuse, even if you have not yet obtained copyright permissions or are unsure of your material's copyright compatibility. Once you have responded and addressed all other outstanding technical requirements, you may resubmit your manuscript within Editorial Manager. 
Potential Copyright Issues:

- Figure 1. Please (a) provide a direct link to the base layer of the map (i.e., the country or region border shape) and ensure this is also included in the figure legend; and (b) provide a link to the terms of use / license information for the base layer image or shapefile. We cannot publish proprietary or copyrighted maps (e.g. Google Maps, Mapquest) and the terms of use for your map base layer must be compatible with our CC BY 4.0 license.
Note: if you created the map in a software program like R or ArcGIS, please locate and indicate the source of the basemap shapefile onto which data has been plotted.
If your map was obtained from a copyrighted source please amend the figure so that the base map used is from an openly available source. Alternatively, please provide explicit written permission from the copyright holder granting you the right to publish the material under our CC BY 4.0 license.
If you are unsure whether you can use a map or not, please do reach out and we will be able to help you. The following websites are good examples of where you can source open access or public domain
* U.S. Geological Survey (USGS) - All maps are in the public domain. (http://www.usgs.gov)
* PlaniGlobe - All maps are published under a Creative Commons license so please cite u201cPlaniGlobe, http://www.planiglobe.com, CC BY 2.0u201d in the image credit after the caption. (http://www.planiglobe.com/?lang=enl)
* Natural Earth - All maps are public domain. (<http://www.naturalearthdata.com/about/terms-of-use/>).

**Authors’answer : Done.**

3) Please amend your detailed Financial Disclosure statement. This is published with the article. It must therefore be completed in full sentences and contain the exact wording you wish to be published.
State the initials, alongside each funding source, of each author to receive each grant. For example: "This work was supported by the National Institutes of Health (####### to AM; ###### to CJ) and the National Science Foundation (###### to AM).".
If you did not receive any funding for this study, please simply state: u201cThe authors received no specific funding for this work.u201d

**Authors’answer : Amended as required.**

**As the authors answered all the Editor and Reviewers remarks and appropriately included these answers in this revised version of the manuscript, they hope that this revised version will be accepted for publication in PNTD.**

**Yours sincerely,**

**Prof. Michel DRANCOURT, MD, PhD.**

**Corresponding author**
