# Supplementary material for: Genomic microdiversity of Mycobacterium africanum in Sénégal
Source: PLoS Negl Trop Dis. 2026 Oct 26;20(10):e0014770. doi: 10.1371/journal.pntd.0014770 (PMC13727044; doi:10.1371/journal.pntd.0014770)
Supplement: Supplementary file 2 [file pntd.0014770.s009.docx]

Dear Editor,

Please find enclosed the second revised version of the manuscript **PNTD-D-25-01817R1** entitled « **Genomic microdiversity of *Mycobacterium africanum* in Sénégal**. » by Joséphine BADIANE and collaborators, along with the point-by-point answers to the Editors and Reviewers.

* A letter that responds to each point raised by the editor and reviewer(s). You should upload this letter as a separate file labeled 'Response to Reviewers'. This file does not need to include responses to any formatting updates and technical items listed in the 'Journal Requirements' section below.

Authors’answer : Done

* A marked-up copy of your manuscript that highlights changes made to the original version. You should upload this as a separate file labeled 'Revised Manuscript with Track Changes'.

Authors’answer : Done

* An unmarked version of your revised paper without tracked changes. You should upload this as a separate file labeled 'Manuscript'.

Authors’answer : Done

**Journal Requirements:**

If the reviewer comments include a recommendation to cite specific previously published works, please review and evaluate these publications to determine whether they are relevant and should be cited. There is no requirement to cite these works unless the editor has indicated otherwise.

1) Please provide an Author Summary. This should appear in your manuscript between the Abstract (if applicable) and the Introduction and should be 150-200 words long. The aim should be to make your findings accessible to a wide audience that includes both scientists and non-scientists. Sample summaries can be found on our website under Submission Guidelines:
https://journals.plos.org/plosntds/s/submission-guidelines#loc-parts-of-a-submission

Authors’answer : The 106-word long Author Summary was already provided in the previous version (lines 66-76).

2) We notice that your supplementary figure 1 is uploaded with the file type 'Figure'. Please amend the file type to 'Supporting Information'. Please ensure that each Supporting Information file has a legend listed in the manuscript after the references list.

Authors’answer: Done

3) Pease ensure to include the direct link in the figure legend for Figure 1."

Authors’answer : The direct link for Figure 1 is already provided in the caption. You need to place the cursor on the link, press Ctrl, and then click.

**Reviewers' Comments:**

Reviewer #1: -Are the objectives of the study clearly articulated with a clear testable hypothesis stated? OK
-Is the study design appropriate to address the stated objectives? OK
-Is the population clearly described and appropriate for the hypothesis being tested? OK
-Is the sample size sufficient to ensure adequate power to address the hypothesis being tested? OK
-Were correct statistical analysis used to support conclusions? OK
-Are there concerns about ethical or regulatory requirements being met? OK

Authors’answer : The authors acknowledge this general positive comment.

Reviewer #2: There are still important flaws in the methodology, and they appear more clearly thanks to supporting information that has been provided as asked during the first round of review.

Authors’answer : The authors will take into consideration all of the detailled comments by reviewer n°2.

**Results**


-Does the analysis presented match the analysis plan?

-Are the results clearly and completely presented?

-Are the figures (Tables, Images) of sufficient quality for clarity?

Reviewer #1: -Does the analysis presented match the analysis plan? OK
-Are the results clearly and completely presented? OK
-Are the figures (Tables, Images) of sufficient quality for clarity? OK

Reviewer #2: only part of the results are reliable.
All figures lack sufficient definition.

Authors’answer : The authors will follow the instructions regarding Figures.

**Conclusions**


-Are the conclusions supported by the data presented?

-Are the limitations of analysis clearly described?

-Do the authors discuss how these data can be helpful to advance our understanding of the topic under study?

-Is public health relevance addressed?

Reviewer #1: -Are the conclusions supported by the data presented? OK
-Are the limitations of analysis clearly described? OK
-Do the authors discuss how these data can be helpful to advance our understanding of the topic under study? OK
-Is public health relevance addressed? OK

Reviewer #2: only approx half of the conclusions are well supported (those linked to TB-Profiler outputs).

Authors’answer : The authors will take into consideration all the reviewer n°2 comments to improve the relevance of the report.

**Editorial and Data Presentation Modifications?**


Use this section for editorial suggestions as well as relatively minor modifications of existing data that would enhance clarity. If the only modifications needed are minor and/or editorial, you may wish to recommend “Minor Revision” or “Accept”.

Reviewer #1: (No Response)

Reviewer #2: (No Response)

**Summary and General Comments**


Use this section to provide overall comments, discuss strengths/weaknesses of the study, novelty, significance, general execution and scholarship. You may also include additional comments for the author, including concerns about dual publication, research ethics, or publication ethics. If requesting major revision, please articulate the new experiments that are needed.

Reviewer #1: The authors have addressed each point in a clear and comprehensive manner. The revisions have significantly improved the clarity and overall quality of manuscript.
I have no further comments or concerns.

Authors’answer : The authors acknowledge this general positive comment.

Reviewer #2: The revised version of the manuscript includes more raw data, as suggested during the first review. However, while specific attention was drawn to a pitfall in analyses, namely regarding the use of de novo assemblies for inferring SNPs presence, no change in methodology was proposed, and the clarification of the methodology and the raw data provided allows to think that several analyses (especially the phylogenetic analysis) are flawed. Added to this are some strange inconsistencies (mention of blastn for genome cleaning, and this only in the result section, use of SNP-sites to extract SNPs, improper reference to the supp table, absence of some supp information). In the end, provided information support only approximately half of the claims of the authors, and irrelevant information regarding methods, incorrect Suppl. Table numbering adds to the weaknesses of the study. Hence, even if the topic of the study is of interest, it seems dangerous to publish this study in its present form, due to the lack of data supporting clear reliability. If another version of the article were to be submitted, it should modify the whole genome analysis, or be restricted to the clear description of phenotypic vs genotypic data and to the outputs of TB-Profiler. Also, special attention should be put on consistency between methods mentioned in the results section and method having actually helped gather results.
Detailed comments:
-l.39 SNP-sites: this tool is not the one that can extract SNPs (it can only reduce the size of an alignment by extracting all positions for which all strains share the same SNP). In addition, also as detailed below, the whole applied procedure, namely reconstructing genomes by de novo assembly using Roary is suboptimal. As detailed in reviews such as Mehan et al, 2019, the authors should have used snippy or the --call_whole_genome argument of tbprofiler, or other all in one tools such as Phyresse or MTBseq

Authors’answer : The authors will take into considerations all the detailed considerations, as below. In accordance with recommendations from the literature, particularly Mehan et al. (2019), we have revised our methodological approach. Specifically, we used TB-Profiler with the --call_whole_genome option, which enables integrated alignment of raw sequencing reads to the H37Rv reference genome, variant calling, and SNP extraction.
We therefore followed the recommended methodology and have revised the manuscript accordingly to clarify this point. We hope that these explanations adequately address your concern.

l.146-150 + l.160-161 : again, as mentioned in reviewer of version 1 of the article, de novo assemblies are suboptimal for extracting SNPs and should have been replaced by TBprofiler --call_whole_genome argument, or by snippy analysis. In addition, the authors then mention sequence alignment. This is strange because once SNPs have been extracted for all strains and combined in a single vcf file, there should be no need for such a step of sequences alignment: the extraction of SNPs from the genome creates artificial pseudo sequence of the same length, where only information at the same position may have a related origin. Destroying this alignment by an additional step can only introduce errors.
The authors argue that de novo assembly is necessary because L5 and L6 genomes include sequences that are absent from H37Rv. They have a point, but such assembly is only reliable when people have obtained long reads sequencing data. In addition, the highest number of additional genes that has been identified in L5 is 34 genes. This is little in comparison to the 4,173 genes consensually predicted. In contrast CheckM analysis focuses solely on 693 genes (see below).

Authors’answer : In particular, we removed the de novo assembly step and instead adopted an approach based on TB-Profiler using the --call_whole_genome option, followed by robust variant calling. The resulting SNPs were then used to generate consensus sequences. The manuscript has been revised accordingly to reflect these methodological changes.

l. 150 : improper syntax, and authors refer to coverage and not depth at this line -> to be changed.

Authors’answer : Corrected accordingly (Line 155).

l.157 : “SNP values” does not make sense. Replace by “SNP-distance values” or “pairwise-comparisons SNP numbers”

Authors’answer : Corrected accordingly (Line 45, 209).

Statistical analyses and results l. 241 and following: there is no clear relevance of comparing the proportion f resistance isolates for different antibiotics.

Authors’answer : The authors do not understand this remark; they do not understand why such (classical / basic) comparisons are irrelevant? *A contrario*, this kind of comparisons fuelled part of the discussion related to the heterogeneity of *M. africanum* (antibiotic resistance being used as a biomarker, not in its derived and important use for treating patients) in Sénégal and geographical relationships.

l.182: genome cleaning using BLASTn makes no sense; in addition, it is not clear what “genome” refers to: assemblies or raw sequencing data? Furthermore, no such thing is described in the methods section.

Authors’answer : The methodology has been thoroughly revised, and all steps have now been clearly and explicitly described. In accordance with the previous comments, we have removed this entire section from the Methods and replaced it with an approach based on consensus sequences generated using TB-Profiler (--call_whole_genome). This change renders the “genome cleaning” step unnecessary.

l. 185: The S1 Table referred to at this line and the subsequent, as available in SUPPLEMENTARY MATERIALS.docx, is entitled “Supplementary Table S1: Comparison of the resistance rate of each antibiotic, with the average resistance rate of all other antibiotics tested.” It is Table S6 that contains CheckM outputs. But the information on the provided output is too scarce to be interpretable. Of note, CheckM focuses on highly conserved genes (apparently 693 genes, out of the more than 4,000 genes in M. tuberculosis H37Rv genome), and not at all on the totality of the genome, which means that Completeness in CheckM output does not relate to real genome completeness but rather to the extent to which highly conserved genes are actually present in the reconstructed genomes.

Authors’answer : The annotation of the supplementary tables has been revised. Following the revision of our methodology, we have completely removed the approach based on de novo assemblies. Consequently, all analyses related to assembly quality assessment, including results obtained with CheckM as well as contamination estimates, have been removed from both the manuscript and the supplementary materials. The revised manuscript no longer contains ambiguities related to the interpretation of genome completeness based on conserved genes. We hope that these modifications fully address the concerns raised.

Subsequent descriptive statistics regarding SNP distance, supposedly available in Table S3, are only available in a very difficult to read format as labels of fig S1. Some values of these SNP distances are well above the expectations (up to 3029) which is highly suggestive of errors in the SNPs identification. Indeed others studies (for instance Guyeux et al, 2024) have detected a maximum interlineage distance of 2,100 SNPs while intra-L6 distance lied below 1,500 SNPs. Altogether, supporting information is still incomplete and some of the supporting information provided actually points out to substantial noise and errors in the analyses. Downstream analyses regarding pairwise distances (l.275 and following) are thus not reliable.

Authors’answer : Following this observation, we completely reanalysed the SNP data in accordance with recommended methodological practices, using TB-Profiler with the --call_whole_genome option. This approach enables genome-wide variant calling. The SNP distances obtained after reanalysis are now consistent with values reported in the literature, particularly those described by Guyeux et al. 2024, with inter-lineage and intra-lineage distances falling within expected thresholds. The manuscript and supplementary files have been thoroughly revised to improve the quality, transparency, and reproducibility of the results. We hope that these modifications address the concerns raised.

Linked to l. 263 and following: while authors claim that sublineage defining positions were manually checked, no related method is described, and the reference to Table S6 (actually most relevant data lie in Table S5) suggest that the only thing authors did is to look thoroughly at this table and not look at reads alignments. Still, the reference to 1 read out of 89 with a sublineage SNP suggests that they may have performed read alignment exploration, but no supporting data is provided. In another version of the article, the underlying method should be made clearly explicit.

Authors’answer : Authors’answer: Since the annotation of the supplementary tables has been revised, this part of the manuscript now correctly refers to supplementary Tables S5 and S6.

We would like to clarify that manual inspection of SNP positions defining sub-lineages was performed by examining read alignments using IGV (http://www.broadinstitute.org/igv/).

This step involved visually assessing mapping quality, coverage at positions of interest, and the consistency of the observed variants, including the proportion of reads supporting each SNP.

l. 188: The expression “215.19% average sequencing coverage” does not make sense. If you use a percentage, it should be clear to what it is referring (i.e. percentage of what?)

Authors’answer : For clarity, these data have been removed, and the assessment of sequencing quality is now referred to in Supplementary Table S4.

l. 193 : lineage assignation: method (using TB-Profiler) should be reminded of.

Authors’answer : Done (line 194) and the version used is specified in the paragraph Bioinformatics analysis line 159

l. 198 : “phylogenetic analysis”/Fig. S1. The figure should have a much better definition to be readable.

Authors’answer : Done

l. 217: Fig. 2: The figure still contains a blank part, as in version 1 of the article.

Authors’answer : Figure 2 has been revised: the empty spaces have been removed, and the resolution has also been improved.

l.220 and following, it should be explained that the detailed analysis of Tb-profiler resistance data was only provided for 19 strains, or rather, this analysis should be extended to all samples. It is very unfortunate that for the 19 samples for which they are available, the phenotypic data are not put side by side with the genotypic resistance data. They should be.

Authors’answer : The detailed analysis using TB Profiler focused on the 16 antibiotic-resistant strains instead of 19. Based on the data summarized in Supplementary Table 5, we attempted to show the depth position and mutation frequency for each mutation predicted by the whole-genome analysis.

Regarding the phenotypic data, whose comparison with the genomic data is illustrated in Table 1, unfortunately only 19 strains were tested using MGIT 960 system. For this reason, the comparison was limited to these strains. As noted by the reviewer, Table 1 presents the genomic and phenotypic data side by side.

The whole-genome sequencing data are shown in the supplementary table 4.

l. L.329 “density of 951 SNPs” makes no sense. Rephrase.

Authors’answer : This sentence has been removed from the text.

Minor comments:
l.29 and l.62 Modify as in the introduction: Senegal is not the country of origin nore the birthplace of M. africanum, but it is indeed the country in which it was first described. It would be relevant in this context to sum up the results of Coscolla et al study (2021)

Authors’answer : Corrected accordingly (Line 29 and 66).

l. 48 confusing phrasing: not clear whether the streptomycin resistant strains is to be counted on the 16/19 isolates or not. It is also not clear whether strains carrying embC mutation Ala307Thr were identified as resistant phenotypically or not.

Authors’answer : For clarity, this section has been revised in accordance with the data presented in Table 1: Antibiotic susceptibility profiling. Whole genome vs DST on nineteen *Mycobacterium africanum* isolates tested with the BACTEC MGIT 960 system.

l. 51: as the data gathered offer a first detailed picture of M. afri diversity in Senegal, it is inappropriate to speak of an “evolving geographic structuration”. What is confirmed is the predominance of L6, and some diversity among L6 strains. Or if the authors want to argue on the evolution of the L presence in Senegal, they should more carefully report the dates of the different references and the trends revealed by their confrontation.

Authors’answer : The reviewer is perfectly right, these sentences have been rephrased, accordingly (Lines 54-55)

The discrepancy between DST and WGS is not necessarily a sign that phenotypic DST is not effective. It could be a sign of difficulty to obtain WGS data actually corresponding to clinical samples due to the multiple steps of data transmission and a suboptimal connexion between samples and labels. The conclusion is thus an overstatement.

Authors’answer : The authors fully disagree with the reviewer that DST discrepancies are most probably due to mishandling of samples along the analytical chain, from the CHU Le Dantec in Dakar to IHU in Marseille:

1) This analytical chain has always been under quality insurance in both centers, both centers being under external quality tests to insure quality

2) In the present work, all the isolates analysed in Marseille were indeed identified as *M. africanum*, as anticipated through results obtained in Dakar

3) In the present work, both DST in Dakar and WGS in Marseille were performed incorporating experimental controls.

More contributively, the authors propose that more works are needed in order to progressively resolve the actual discrepancies (observed in all mycobacteriology reference centers in the World) between DST and WGS; as suggested in line 57 in the revised Abstract.

l. 63 : Correct “the majority of tuberculosis cases” for a significant part of tuberculosis cases, as it was only shown for Guinea-Bissau that M. africanum contributed to more than 40% of TB cases.

Authors’answer : Corrected accordingly (Line 68).

l.186 spolygotyping -> spoligotyping

Authors’answer : Corrected accordingly (Line 190).

**Figure resubmission:**

While revising your submission, we strongly recommend that you use PLOS’s NAAS tool (https://ngplosjournals.pagemajik.ai/artanalysis) to test your figure files. NAAS can convert your figure files to the TIFF file type and meet basic requirements (such as print size, resolution), or provide you with a report on issues that do not meet our requirements and that NAAS cannot fix.

After uploading your figures to PLOS’s NAAS tool - https://ngplosjournals.pagemajik.ai/artanalysis, NAAS will process the files provided and display the results in the "Uploaded Files" section of the page as the processing is complete. If the uploaded figures meet our requirements (or NAAS is able to fix the files to meet our requirements), the figure will be marked as "fixed" above. If NAAS is unable to fix the files, a red "failed" label will appear above. When NAAS has confirmed that the figure files meet our requirements, please download the file via the download option, and include these NAAS processed figure files when submitting your revised manuscript.

Authors’answer : The figures have been revised in accordance with the journal’s suggestions, using “https://ngplosjournals.pagemajik.ai/artanalysis”

As the authors answered all the Editors’ and Reviwers’comments and corrected the manuscript accordingly, they hope that this revised version will be accepted for publication in PNTD.

Yours sincerely,

Prof. Michel DRANCOURT, MD, PhD

Corresponding author
