# Supplementary material for: Genomic microdiversity of Mycobacterium africanum in Sénégal
Source: PLoS Negl Trop Dis. 2026 Oct 26;20(10):e0014770. doi: 10.1371/journal.pntd.0014770 (PMC13727044; doi:10.1371/journal.pntd.0014770)
Supplement: Supplementary file 3 [file pntd.0014770.s010.docx]

PNTD-D-25-01817R3
Genomic microdiversity of Mycobacterium africanum in Sénégal.
PLOS Neglected Tropical Diseases

Dear Editor,

Please find enclosed the third, revised version of the manuscript entitled « Genomic microdiversity of Mycobacterium africanum in Sénégal » (PNTD-D-25-01817R 3) along with the answers of the authors to the reviewer’s comments.

Please include the following items when submitting your revised manuscript:

* A letter that responds to each point raised by the editor and reviewer(s). You should upload this letter as a separate file labeled 'Response to Reviewers'. This file does not need to include responses to any formatting updates and technical items listed in the 'Journal Requirements' section below.

**Authors’answer : Enclosed.**

* A marked-up copy of your manuscript that highlights changes made to the original version. You should upload this as a separate file labeled 'Revised Manuscript with Track Changes'.

**Authors’answer : Enclosed.**

* An unmarked version of your revised paper without tracked changes. You should upload this as a separate file labeled 'Manuscript'.

**Authors’answer : Enclosed.**

**Reviewers' Comments:**

Reviewer #2: Methods used to describe diversity still rely on strange proxies
l.299: median minus 2 std dev used extensively as a proxy for diversity... but:
*std dev is defined according to the mean, so if combined to Something, this should be the mean.*if one wants to capture the global trend of a sample for a quantitative parameter, mean is chosen when this parameter is normally distribued, and median is preferred otherwise. Mean minus 2 std dev identifies the threshold for 2,5% of strains with the lowest diversity in the subsample, and does not capture any global trend

**Authors’answer : The reviewer is perfectly right, this section of the manuscript has been revised: standard deviation is now reported alongside the mean, and median and interquartile range (IQR) have been calculated to assess the distribution of the strains and results are reported in Lines 37-38 (Abstract) and lines 170-179 (Text).**

*a standard value used for diversity assessment is the presence of strains with less than 12 SNPs distance, suggestive of relatively recent transmission, in accordance with standards where recent transmission was independently supported (Walker et al, 2013, for instance). There is no mention of this standard here

**Authors’answer : The reviewer is right, this tabulation is now reported in the M&M section (lines 177-179), the result in lines 274-276 and the discussion 339-341. In fact, we observed no pair of isolates exhibited less than 12 SNPs.**

Methods regarding resistance prediction suffer from flaws: Many mutations described as responsible for resistance in the text and tables are in fact annotated as "Uncertain significance" in the WHO catalog. Especially those present in the supposedly MDR strain, and those linked to suppsed ethambutol resistance, that contradict phenotypic results. In fact, as such, these should be considered as not conferring resistance. Please revisit all resistance annotations in light with WHO catalog of such resistance mutations, and annotate in light of their significance or withdraw all mutations that are not clearly associated with resistance.

**Authors’answer : The reviewer is right and according to this remark, all detected mutations were reviewed against the WHO catalogue of mutations. (revised M&M section, lines 164-169; result section, lines 215-224. Consequently, the previous version of comparative mutation analyses and Supplementary Tables S5 and S6, have been deleted from the revised version of the manuscript.**

Methods regarding genome inferences are still partly flawed: the authors should be aware that the genomes they reconstructed through their bioinformatical analyses are faked, copying H37Rv genome and replacing only the ny where SNPs or indels have been identified. This type of faked genome is highly useful for phylogeny, but should not be used and presented as real : no exploration has been made of the number and position of IS6110, deletion regions, etc. Hence the genome sizes reported in S1 table should be withdrawn. Accordingly, there is no relevance to present dDDH data on such genomes. This information should also be withdrawn for result section.

**Authors’answer : The reviewer is perfectly right, and accordingly the genome size values have been deleted from the revised Results section and from the revised Table S1.**

-it is not clear whether SNPs used for phylogeny etc include PE-PPE or not, and other régions possibly associated with imperfect read alignment (see again Meehan 2019). Please describe more precisely.

**Authors’answer : We thank the reviewer for this comment. SNPs used for phylogenetic analyses were filtered to retain only high-confidence variants. In addition, SNPs located in PE/PPE genes and other genomic regions associated with unreliable read alignment, as described by Meehan et al. (2019), were excluded using a masking approach. We have clarified this point in the Methods section line 145-149 and line 156-159.**

Reviewer #2: some data do not match : SN01 coverage indicated to be 41 in S1 table, and 21 in S4 table. All compiled data should be re-checked.

**Authors’answer : This version of the Table S4 was reporting the coverage of mutation regions only, complementary to the Table S1. However, according to above remark by the reviewer, the Table S4 has been revised.** **The mutations highlighted in bold in the table are non-synonymous mutations that may confer antibiotic resistance.**

problems related to methodology as mentionned above.
the long presentation of % of resistance with so small sample sizes is still misleading and of relatively low benefit. And even more misleading considering that most are not resistant at all. This part could be reduced to the mention of mutations observed in several samples, and their potential impact.

**Authors’answer : This reviewer comment is acknowledged and approved. Considering the interpretation of mutations based on the WHO catalogue, this part of the manuscript has been removed, as well as illustrating Supplementary Tables S5 and S6.**

Reviewer #2: Conclusions regarding diversity are well supported.
Conclusions regarding resistance should be re-examined.

**Authors’answer : Done, accordingly.**

**Editorial and Data Presentation Modifications?**


Use this section for editorial suggestions as well as relatively minor modifications of existing data that would enhance clarity. If the only modifications needed are minor and/or editorial, you may wish to recommend “Minor Revision” or “Accept”.

Reviewer #2: Some English writing such as
l. 344 : small sample size (not "effective")

**Authors’answer : Corrected accordingly (line 375).**

Reviewer #2: The article by Drancourt rev.2 et al has been efficiently purged from major biases included in previous versions. It now presents a phylogeny of Mycobacterium africanum isolates identified in Senegal that seems reliable. The description of their geographic distribution is backed up by a clear map presenting both population sizes and diversity. Information on coverage, reads, is now much clearer and precise.
The disappearance of major defects revealed some other major points that should imperatively be adressed before publication:

Major comments:
-Results describing diversity still rely on strange proxies
l.299: median minus 2 std dev used extensively as a proxy for diversity... but:
*std dev is defined according to the mean, so if combined to Something, this should be the mean.
*if one wants to capture the global trend of a sample for a quantitative parameter, mean is chosen when this parameter is normally distribued, and median is preferred otherwise. Mean minus 2 std dev identifies the threshold for 2,5% of strains with the lowest diversity in the subsample, and does not capture any global trend
*a standard value used for diversity assessment is the presence of strains with less than 12 SNPs distance, suggestive of relatively recent transmission, in accordance with standards where recent transmission was independently supported (Walker et al, 2013, for instance). There is no mention of this standard here
Hence the results on diversity need to be revised.

**Authors’answer : See above.**

-some data do not match : SN01 coverage indicated to be 41 in S1 table, and 21 in S4 table. All compiled data should be re-checked.

**Authors’answer : This version of the Table S4 was reporting the coverage of mutation regions only, complementary to the Table S1. However, according to above remark by the reviewer, the Table S4 has been revised.** **The mutations highlighted in bold in the table are non-synonymous mutations that may confer antibiotic resistance.**

-Many mutations described as responsible for resistance in the text and tables are in fact annotated as "Uncertain significance" in the WHO catalog. Especially those present in the supposedly MDR strain, and those linked to suppsed ethambutol resistance, that contradict phenotypic results. In fact, as such, these should be considered as not conferring resistance. Please revisit all resistance annotations in light with WHO catalog of such resistance mutations, and annotate in light of their significance or withdraw all mutations that are not clearly associated with resistance. This should reshape discussion.

**Authors’answer : Agreed, see above.**

-the authors should be aware that the genomes they reconstructed through their bioinformatical analyses are faked, copying H37Rv genome and replacing only the ny where SNPs or indels have been identified. This type of faked genome is highly useful for phylogeny, but should not be used and presented as real : no exploration has been made of the number and position of IS6110, deletion regions, etc. Hence the genome sizes reported in S1 table should be withdrawn.Accordingly, there is no relevance to present dDDH data on such genomes. This information should also be withdrawn for result section.

**Authors’answer : Agreed, see above.**


Minor points:
-the long presentation of % of resistance with so small sample sizes is still misleading and of relatively low benefit. And even more misleading considering that most are not resistant at all. This part could be reduced to the mention of mutations observed in several samples, and their potential impact.
-l. 344 : small sample size (not "effective")

**Authors’answer : Corrected accordingly, see above.**

-it is not clear whether SNPs used for phylogeny etc include PE-PPE or not, and other régions possibly associated with imperfect read alignment (see again Meehan 2019). Please describe more precisely.
-mutation frequency absent from table S1

**Authors’answer : The mutation frequencies have now been added to Supplementary Table S1.**

-the high contamination and low read number supporting resistance in SN48 should be more clearly mentionned in the text.

**Authors’answer : Thank you for this comment. We agree that the limitations associated with isolate SN48 should be highlighted more clearly. Although the isolate showed an overall median coverage of 87×, the resistance-associated mutations were detected at relatively low allele frequencies, particularly rpoB_p.Ser441Ala (0.15) and katG_p.Ser140Asn (0.16). Therefore, the resistance profile inferred for SN48 should be interpreted with caution. This was confirmed through mutation analysis based on the WHO Catalogue of Mutations Associated with Drug Resistance and has been clarified in the Results section entitled "WGS-based antibiotic susceptibility profiling". (See Lines 225-227).**

**Figure resubmission:**

While revising your submission, we strongly recommend that you use PLOS’s NAAS tool (https://ngplosjournals.pagemajik.ai/artanalysis) to test your figure files. NAAS can convert your figure files to the TIFF file type and meet basic requirements (such as print size, resolution) or provide you with a report on issues that do not meet our requirements and that NAAS cannot fix.

After uploading your figures to PLOS’s NAAS tool - https://ngplosjournals.pagemajik.ai/artanalysis, NAAS will process the files provided and display the results in the "Uploaded Files" section of the page as the processing is complete. If the uploaded figures meet our requirements (or NAAS is able to fix the files to meet our requirements), the figure will be marked as "fixed" above. If NAAS is unable to fix the files, a red "failed" label will appear above. When NAAS has confirmed that the figure files meet our requirements, please download the file via the download option, and include these NAAS processed figure files when submitting your revised manuscript.

**Reproducibility:**

To enhance the reproducibility of your results, we recommend that authors of applicable studies deposit laboratory protocols in protocols.io, where a protocol can be assigned its own identifier (DOI) such that it can be cited independently in the future. Additionally, PLOS ONE offers an option to publish peer-reviewed clinical study protocols. Read more information on sharing protocols at https://plos.org/protocols?utm_medium=editorial-email&utm_source=authorletters&utm_campaign=protocols

**The authors did this best to address all the final remarks by the reviewer n°2, they did revise the manuscript accordingly and they hope that this third revised version will be accepted for publication.**

**Prof. M. DRANCOURT**

**Corresponding author.**
