# Supplementary material for: Genomic microdiversity of Mycobacterium africanum in Sénégal
Source: PLoS Negl Trop Dis. 2026 Oct 26;20(10):e0014770. doi: 10.1371/journal.pntd.0014770 (PMC13727044; doi:10.1371/journal.pntd.0014770)
Supplement: Supplementary file 4 [file pntd.0014770.s011.docx]

PNTD-D-25-01817R4
Genomic microdiversity of Mycobacterium africanum in Sénégal.
PLOS Neglected Tropical Diseases

Dear Editor,

Please find enclosed the third, revised version of the manuscript entitled « Genomic microdiversity of Mycobacterium africanum in Sénégal » (PNTD-D-25-01817R4) along with the answers of the authors to the reviewer comments.

* A letter that responds to each point raised by the editor and reviewer(s). You should upload this letter as a separate file labeled 'Response to Reviewers'. This file does not need to include responses to any formatting updates and technical items listed in the 'Journal Requirements' section below.

**Authors’answer : Enclosed.**

* A marked-up copy of your manuscript that highlights changes made to the original version. You should upload this as a separate file labeled 'Revised Manuscript with Track Changes'.

**Authors’answer : Enclosed.**

* An unmarked version of your revised paper without tracked changes. You should upload this as a separate file labeled 'Manuscript'.

**Authors’answer : Enclosed.**

**Reviewers' Comments:**

lane 71: the fact that L5 is present in Gambia and rarely in Senegal (Lane 71-72) is not evident to the reviewer. Please add reference

**Authors’answer : The authors acknowledge the reviewer’s concerns and agree that this point required further clarification. Accordingly, this section of the manuscript has been carefully revised and corrected in accordance with the relevant scientific literature. As observed in Sénégal, lineage L5 appears to be rarely detected in The Gambia (lines 363-371).**

lane 107: reference for the CTAB method is missing, please add reference.

**Authors’answer : Done line 109**

lane 229: one space is missing in "DSTin 16". it should be : "DST in 16"

**Authors’answer : Done, line 231**

As far as I'm concerned, the main conclusion of this study is that L5 is virtually absent from Senegal, whereas it is present (according to the authors) in The neighboring Gambia, which seems—at least at first glance—to share quite a few similarities with Senegal. This could be discussed in a conclusive sentence.

**Authors’answer : Contrary to what was stated in version R3, The Gambia, like Sénégal and several other West African countries, shows a predominance of lineage L6, with lineage L5 being rarely detected. A few concluding sentences have been added to the manuscript to clarify this point (lines 363-371).**

**The authors did this best to address all the final remarks by the reviewer n°3, they did revise the manuscript accordingly and they hope that this fourth revised version will be accepted for publication.**

**Prof. M. CAMARA,**

**Corresponding author.**
