# Supplementary material for: Genomic microdiversity of Mycobacterium africanum in Sénégal
Source: PLoS Negl Trop Dis. 2026 Oct 26;20(10):e0014770. doi: 10.1371/journal.pntd.0014770 (PMC13727044; doi:10.1371/journal.pntd.0014770)
Supplement: Peer review history

# Peer review: Decision Letter 0

**DOI:** 10.1371/journal.pntd.0014770.r001

**Authors:** Mathieu Picardeau

3 Dec 2025

PNTD-D-25-01817

Genomic microdiversity of Mycobacterium africanum in Sénégal.

PLOS Neglected Tropical Diseases

Dear Dr. Drancourt,

Thank you for submitting your manuscript to PLOS Neglected Tropical Diseases. After careful consideration, we feel that it has merit but does not fully meet PLOS Neglected Tropical Diseases's publication criteria as it currently stands. Therefore, we invite you to submit a revised version of the manuscript that addresses the points raised during the review process.

Please submit your revised manuscript within by Feb 01 2026 11:59PM. If you will need more time than this to complete your revisions, please reply to this message or contact the journal office at plosntds@plos.org. When you're ready to submit your revision, log on to https://www.editorialmanager.com/pntd/ and select the 'Submissions Needing Revision' folder to locate your manuscript file.

Please include the following items when submitting your revised manuscript:

* A rebuttal letter that responds to each point raised by the editor and reviewer(s). You should upload this letter as a separate file labeled 'Response to Reviewers'. This file does not need to include responses to any formatting updates and technical items listed in the 'Journal Requirements' section below.

* A marked-up copy of your manuscript that highlights changes made to the original version. You should upload this as a separate file labeled 'Revised Manuscript with Track Changes'.

* An unmarked version of your revised paper without tracked changes. You should upload this as a separate file labeled 'Manuscript'.

If you would like to make changes to your financial disclosure, competing interests statement, or data availability statement, please make these updates within the submission form at the time of resubmission. Guidelines for resubmitting your figure files are available below the reviewer comments at the end of this letter.

We look forward to receiving your revised manuscript.

Kind regards,

Mathieu Picardeau

Section Editor

PLOS Neglected Tropical Diseases

Shaden Kamhawi

co-Editor-in-Chief

PLOS Neglected Tropical Diseases

orcid.org/0000-0003-4304-636XX

Paul Brindley

co-Editor-in-Chief

PLOS Neglected Tropical Diseases

orcid.org/0000-0003-1765-0002

**Journal Requirements:**

1) Please upload all main figures as separate Figure files in .tif or .eps format. For more information about how to convert and format your figure files please see our guidelines:

https://journals.plos.org/plosntds/s/figures

2) Some material included in your submission may be copyrighted. According to PLOSu2019s copyright policy, authors who use figures or other material (e.g., graphics, clipart, maps) from another author or copyright holder must demonstrate or obtain permission to publish this material under the Creative Commons Attribution 4.0 International (CC BY 4.0) License used by PLOS journals. Please closely review the details of PLOSu2019s copyright requirements here: PLOS Licenses and Copyright. If you need to request permissions from a copyright holder, you may use PLOS's Copyright Content Permission form.

Please respond directly to this email and provide any known details concerning your material's license terms and permissions required for reuse, even if you have not yet obtained copyright permissions or are unsure of your material's copyright compatibility. Once you have responded and addressed all other outstanding technical requirements, you may resubmit your manuscript within Editorial Manager.

Potential Copyright Issues:

- Figure 1. Please (a) provide a direct link to the base layer of the map (i.e., the country or region border shape) and ensure this is also included in the figure legend; and (b) provide a link to the terms of use / license information for the base layer image or shapefile. We cannot publish proprietary or copyrighted maps (e.g. Google Maps, Mapquest) and the terms of use for your map base layer must be compatible with our CC BY 4.0 license.

Note: if you created the map in a software program like R or ArcGIS, please locate and indicate the source of the basemap shapefile onto which data has been plotted.

If your map was obtained from a copyrighted source please amend the figure so that the base map used is from an openly available source. Alternatively, please provide explicit written permission from the copyright holder granting you the right to publish the material under our CC BY 4.0 license.

If you are unsure whether you can use a map or not, please do reach out and we will be able to help you. The following websites are good examples of where you can source open access or public domain maps:

* U.S. Geological Survey (USGS) - All maps are in the public domain. (http://www.usgs.gov)

* PlaniGlobe - All maps are published under a Creative Commons license so please cite u201cPlaniGlobe, http://www.planiglobe.com, CC BY 2.0u201d in the image credit after the caption. (http://www.planiglobe.com/?lang=enl)

* Natural Earth - All maps are public domain. (http://www.naturalearthdata.com/about/terms-of-use/).

3) Please amend your detailed Financial Disclosure statement. This is published with the article. It must therefore be completed in full sentences and contain the exact wording you wish to be published.

State the initials, alongside each funding source, of each author to receive each grant. For example: "This work was supported by the National Institutes of Health (####### to AM; ###### to CJ) and the National Science Foundation (###### to AM).".

If you did not receive any funding for this study, please simply state: u201cThe authors received no specific funding for this work.u201d

**Reviewers' Comments:**

Reviewer's Responses to Questions

**Key Review Criteria Required for Acceptance?**

As you describe the new analyses required for acceptance, please consider the following:

**Methods**

-Are the objectives of the study clearly articulated with a clear testable hypothesis stated?

-Is the study design appropriate to address the stated objectives?

-Is the population clearly described and appropriate for the hypothesis being tested?

-Is the sample size sufficient to ensure adequate power to address the hypothesis being tested?

-Were correct statistical analysis used to support conclusions?

-Are there concerns about ethical or regulatory requirements being met?

Reviewer #1: 1. Are the objectives of the study clearly articulated with a clear testable hypothesis stated?

Yes. The objective—to describe the genomic diversity of Mycobacterium africanum in Senegal using WGS and compare phenotypic and genotypic resistance—is clearly stated in the Introduction. While the hypothesis is implicit rather than explicitly formulated, the aim is testable.

2. Is the study design appropriate to address the stated objectives?

Yes. A retrospective design using isolates collected during routine surveillance is appropriate for assessing genomic diversity and resistance patterns.

3. Is the population clearly described and appropriate for the hypothesis being tested?

Partially. The manuscript specifies that isolates were obtained from eight regions in Senegal between 2010 and 2015, but it does not detail the type of clinical specimens or patient characteristics. This limits epidemiological context.

4. Is the sample size sufficient to ensure adequate power to address the hypothesis being tested?

For a descriptive genomic study, 51 isolates provide meaningful insights into diversity, though the authors acknowledge limitations for regional comparisons. For statistical comparisons of resistance rates, the sample size is modest but acceptable for exploratory analysis.

5. Were correct statistical analyses used to support conclusions?

Yes. The manuscript uses binomial proportion tests and McNemar’s test with Benjamini–Hochberg correction for multiple comparisons, which are appropriate for the data presented.

6. Are there concerns about ethical or regulatory requirements being met?

No major concerns. The study uses anonymized retrospective isolates collected during routine care, and the authors state compliance with the Declaration of Helsinki. No patient identifiers or sensitive data were included.

Reviewer #2: objectives clear, sample size ok

inappropriate use of assemblies, poor statistical description of data

**********

**Results**

-Does the analysis presented match the analysis plan?

-Are the results clearly and completely presented?

-Are the figures (Tables, Images) of sufficient quality for clarity?

Reviewer #1: 1. Does the analysis presented match the analysis plan?

Yes. The results follow the described methods: WGS for lineage and SNP analysis, DST for 19 isolates, and statistical comparisons of resistance rates.

2. Are the results clearly and completely presented?

Yes. The main findings are well described.

3. Are the figures (Tables, Images) of sufficient quality for clarity?

Partially. Figures 1 and 2 are informative but could benefit from additional details (see general comments). Figure 3 is difficult to read and should be improved for resolution and clarity.

Reviewer #2: results incomplete (info on coverage for all samples)

possibly manually modified figure (Fig. 2)

**********

**Conclusions**

-Are the conclusions supported by the data presented?

-Are the limitations of analysis clearly described?

-Do the authors discuss how these data can be helpful to advance our understanding of the topic under study?

-Is public health relevance addressed?

Reviewer #1: 1. Are the conclusions supported by the data presented?

Yes. The conclusions about genomic diversity, lineage distribution, and emerging resistance are consistent with the WGS and DST data presented.

2. Are the limitations of analysis clearly described?

Partially. The discussion acknowledges geographic bias (Dakar overrepresented) and small sample size for some regions, but does not explicitly address the lack of temporal data or clinical specimen details.

3. Do the authors discuss how these data can be helpful to advance our understanding of the topic under study?

Yes. The authors emphasize the importance of genomic surveillance and suggest a “One Health” approach for future research, which advances understanding of M. africanum epidemiology.

4. Is public health relevance addressed?

Yes. The manuscript highlights implications for tuberculosis control in Senegal and West Africa, particularly regarding drug resistance prediction and surveillance.

Reviewer #2: Global conclusions such as L6 prevalence as compared to L5 supported.

discssion interesting regarding ecology of the bacteria

public Health relevant

**********

**Editorial and Data Presentation Modifications?**

Use this section for editorial suggestions as well as relatively minor modifications of existing data that would enhance clarity. If the only modifications needed are minor and/or editorial, you may wish to recommend “Minor Revision” or “Accept”.

Reviewer #1: Several points could be clarified or improved:

1. Clarification of isolate selection: The manuscript states that 51 isolates were analyzed from a pool of 549 samples collected between 2010 and 2015. It is unclear whether these 51 isolates represent all M. africanum isolates identified during this period or a subset selected based on specific criteria. Please clarify the selection strategy and whether the sampling was exhaustive or biased.

2. Source of clinical samples and temporal distribution: The manuscript does not specify the type of clinical specimens from which the isolates were obtained. This information is important for understanding the epidemiological context. Additionally, providing the temporal distribution of isolates across centers would allow readers to relate the evolution of mutations (SNPs) to time and geography.

3. Justification for AST on only 19 isolates: AST was performed on 19 out of 51 isolates, but no rationale is provided for this limited testing. Please explain why AST was not performed on all isolates.

4. Presentation of AST Results: Detailed AST results are currently relegated to supplementary tables. Given their importance for clinical and epidemiological interpretation, a summary table comparing phenotypic AST and WGS predictions should be included in the main manuscript.

5. Figure 1: Figure 1 shows sublineage distribution but does not indicate the number of isolates per region. Adding these counts would improve clarity and allow readers to better interpret the geographic structuring.

6. Figure 3:The historical distribution map is interesting but difficult to read. Consider improving resolution and enhancing the legend for better interpretability.

Reviewer #2: (No Response)

**********

**Summary and General Comments**

Use this section to provide overall comments, discuss strengths/weaknesses of the study, novelty, significance, general execution and scholarship. You may also include additional comments for the author, including concerns about dual publication, research ethics, or publication ethics. If requesting major revision, please articulate the new experiments that are needed.

Reviewer #1: This manuscript by Ba Dialo et al. addresses an important and underexplored topic: the genomic diversity of Mycobacterium africanum in Senegal, its country of origin. The study is well-conducted, combining whole genome sequencing (WGS) and phenotypic antimicrobial susceptibility testing (AST) to provide novel insights into lineage distribution and antimicrobial resistance. The work is original, as no previous WGS-based study has characterized M. africanum isolates from Senegal. The findings have significant implications for tuberculosis surveillance and control in West Africa.

Reviewer #2: The article by Diallo et al describes the genomic diversity of Mycobacterium africanum in Senegal using Whole genome sequencing data. This type of study is important as Mtb diversity in this country has been mainly described using genetic footprints from CRISPR region (spoligotyping). The study uses Illumina sequencing on 51 DNA extracts. It confirms that L6 is the major lineage belonging to M. africanum species, but documents for the first time the presence of rare L5 strains (n=1 in Senegal). It also confirms the presence of several L6 sublineages circulating in the country. Antibiotic resistance profiles are derived from TB-Profiler analysis. The distribution of M. africanum diversity is commented in face of the ecological context of strains, proposing hypotheses to account for intriguingly high resistance level to ethambutol. The article is globally sound and well written. However, many imprecisions weaken the reliability of the conclusions.

Major comments:

-the information derived from each bioinformatical analyses is unclear. The way that lineages and antibiotic resistance are informed resembles TB-Profiler outputs but the authors suggest that it derives from the genome assemblies they constructed. TB-Profiler is meant to process directly short-reads sequencing data and assemblies derived from short reads. So, a good practice would be to use TB-Profiler to interpret lineage and antibiotic resistance. So better explanations on what has been done is needed, and rigorous choices on methodology are required. In addition, statistical data retrieved from TB-Profiler need to be presented extensively. A key information is the depth of sequencing. This information is needed for all strains and not only resistant ones. In addition, when mean depth (referred to as mean coverage) is low, a manual exploration of the alignment is crucial to confirm or reject SNP presence. This should be done

-assemblies could help exploring the quality of the data and other features such as the presence of specific regions present in L5 or L6 but absent from other lineages. Yet authors should be cautious about the quality of their assemblies as they harbour coverages (the total length of contigs as compared to H37Rv length) well above the expectation (expectation is less than 110%). Its is likely that in the current assemblies, several sequences are present in multiple copies where only one exists in the genomes. In turn, the samples could be contaminated, which could also lead to false variant inferences. Hence a more extensive presentation of the outputs and/or a more thorough use of these results are needed.

-Fig. 2 tree has a hidden part, and visible shapes seem incompatible. It looks like a manually modified figures which casts doubt on its scientific reliability…

-additional insight should be provided as per how much the gathered data conform, modify or refine L6 history of diversification proposed by Coscolla et al. Of note, in that study, Senegal is not presented as the origin of L6. This point should be amended and/or better discussed.

-the authors made the choice of declaring a strain resistant as soon as some reads carry a resistance mutation. It is important to consider and discuss in the article whether some little contamination with another strain could account for these rare reads carrying the resistance.

Additional comments:

-some information describing the relative prevalence of M. africanum as compared to other lineages would increase the interest of the study

-S1 figure. Clarify the identity of the outgroup.

-Nature of L6 unclassified should be discussed: when they group with a strain of an identified sublineage on the phylogenetic reconstruction, with a branch with strong bootstrap support, it is parsimonious to infer that they belong to the same sublineage. Their not carrying the classifying SNP can be due either to the corresponding SNP not been identified because of low depth at the corresponding position, or to the strain being a mixture of several strains from distinct sublineages so that no sublineage SNP has a sufficient prevalence to call the corresponding classifying SNP, or to the SNP(s) used for classification being inaccurate, allowing only to classify part of the population and/or misclassifying some strains carrying the SNP associated to the sublineage but due to an independent mutation (homoplasy). To make this clear, manual exploration of the alignments of each unclassified sample at classifying SNP positions is required.

**********

PLOS authors have the option to publish the peer review history of their article (what does this mean?). If published, this will include your full peer review and any attached files.

If you choose “no”, your identity will remain anonymous but your review may still be made public.

**Do you want your identity to be public for this peer review?**  For information about this choice, including consent withdrawal, please see our Privacy Policy.

Reviewer #1: No

Reviewer #2: No

[NOTE: If reviewer comments were submitted as an attachment file, they will be attached to this email and accessible via the submission site. Please log into your account, locate the manuscript record, and check for the action link "View Attachments". If this link does not appear, there are no attachment files.]

**Figure resubmission:**

While revising your submission, we strongly recommend that you use PLOS’s NAAS tool (https://ngplosjournals.pagemajik.ai/artanalysis) to test your figure files. NAAS can convert your figure files to the TIFF file type and meet basic requirements (such as print size, resolution), or provide you with a report on issues that do not meet our requirements and that NAAS cannot fix.

After uploading your figures to PLOS’s NAAS tool - https://ngplosjournals.pagemajik.ai/artanalysis, NAAS will process the files provided and display the results in the "Uploaded Files" section of the page as the processing is complete. If the uploaded figures meet our requirements (or NAAS is able to fix the files to meet our requirements), the figure will be marked as "fixed" above. If NAAS is unable to fix the files, a red "failed" label will appear above. When NAAS has confirmed that the figure files meet our requirements, please download the file via the download option, and include these NAAS processed figure files when submitting your revised manuscript.

**Reproducibility:**

To enhance the reproducibility of your results, we recommend that authors of applicable studies deposit laboratory protocols in protocols.io, where a protocol can be assigned its own identifier (DOI) such that it can be cited independently in the future. Additionally, PLOS ONE offers an option to publish peer-reviewed clinical study protocols. Read more information on sharing protocols at https://plos.org/protocols?utm_medium=editorial-email&utm_source=authorletters&utm_campaign=protocols

# Author comment: Author response to Decision Letter 1

**DOI:** 10.1371/journal.pntd.0014770.r002

28 Jan 2026

Attachment Submitted filename: Rebuttal letter 11.01.26.docx

# Peer review: Decision Letter 1

**DOI:** 10.1371/journal.pntd.0014770.r003

**Authors:** Mathieu Picardeau

2 Mar 2026

PNTD-D-25-01817R1

Genomic microdiversity of Mycobacterium africanum in Sénégal.

PLOS Neglected Tropical Diseases

Dear Dr. Drancourt,

Thank you for submitting your manuscript to PLOS Neglected Tropical Diseases. After careful consideration, we feel that it has merit but does not fully meet PLOS Neglected Tropical Diseases's publication criteria as it currently stands. Therefore, we invite you to submit a revised version of the manuscript that addresses the points raised during the review process.

Please submit your revised manuscript within by Apr 01 2026 11:59PM. If you will need more time than this to complete your revisions, please reply to this message or contact the journal office at plosntds@plos.org. When you're ready to submit your revision, log on to https://www.editorialmanager.com/pntd/ and select the 'Submissions Needing Revision' folder to locate your manuscript file.

Please include the following items when submitting your revised manuscript:

* A letter that responds to each point raised by the editor and reviewer(s). You should upload this letter as a separate file labeled 'Response to Reviewers'. This file does not need to include responses to any formatting updates and technical items listed in the 'Journal Requirements' section below.

* A marked-up copy of your manuscript that highlights changes made to the original version. You should upload this as a separate file labeled 'Revised Manuscript with Track Changes'.

* An unmarked version of your revised paper without tracked changes. You should upload this as a separate file labeled 'Manuscript'.

If you would like to make changes to your financial disclosure, competing interests statement, or data availability statement, please make these updates within the submission form at the time of resubmission. Guidelines for resubmitting your figure files are available below the reviewer comments at the end of this letter.

We look forward to receiving your revised manuscript.

Kind regards,

Mathieu Picardeau

Section Editor

PLOS Neglected Tropical Diseases

Mathieu Picardeau

Section Editor

PLOS Neglected Tropical Diseases

Shaden Kamhawi

co-Editor-in-Chief

PLOS Neglected Tropical Diseases

orcid.org/0000-0003-4304-636XX

Paul Brindley

co-Editor-in-Chief

PLOS Neglected Tropical Diseases

orcid.org/0000-0003-1765-0002

**Journal Requirements:**

If the reviewer comments include a recommendation to cite specific previously published works, please review and evaluate these publications to determine whether they are relevant and should be cited. There is no requirement to cite these works unless the editor has indicated otherwise.

1) Please provide an Author Summary. This should appear in your manuscript between the Abstract (if applicable) and the Introduction, and should be 150-200 words long. The aim should be to make your findings accessible to a wide audience that includes both scientists and non-scientists. Sample summaries can be found on our website under Submission Guidelines:

https://journals.plos.org/plosntds/s/submission-guidelines#loc-parts-of-a-submission

2) We notice that your supplementary figure 1 is uploaded with the file type 'Figure'. Please amend the file type to 'Supporting Information'. Please ensure that each Supporting Information file has a legend listed in the manuscript after the references list.

3) Pease ensure to include the direct link in the figure legend for Figure 1."

**Reviewers' Comments:**

Reviewer's Responses to Questions

**Key Review Criteria Required for Acceptance?**

As you describe the new analyses required for acceptance, please consider the following:

**Methods**

-Are the objectives of the study clearly articulated with a clear testable hypothesis stated?

-Is the study design appropriate to address the stated objectives?

-Is the population clearly described and appropriate for the hypothesis being tested?

-Is the sample size sufficient to ensure adequate power to address the hypothesis being tested?

-Were correct statistical analysis used to support conclusions?

-Are there concerns about ethical or regulatory requirements being met?

Reviewer #1: -Are the objectives of the study clearly articulated with a clear testable hypothesis stated? OK

-Is the study design appropriate to address the stated objectives? OK

-Is the population clearly described and appropriate for the hypothesis being tested? OK

-Is the sample size sufficient to ensure adequate power to address the hypothesis being tested? OK

-Were correct statistical analysis used to support conclusions? OK

-Are there concerns about ethical or regulatory requirements being met? OK

Reviewer #2: There are still important flaws in the methodology, and they appear more clearly thanks to supporting information that has been provided as asked during the first round of review.

**********

**Results**

-Does the analysis presented match the analysis plan?

-Are the results clearly and completely presented?

-Are the figures (Tables, Images) of sufficient quality for clarity?

Reviewer #1: -Does the analysis presented match the analysis plan? OK

-Are the results clearly and completely presented? OK

-Are the figures (Tables, Images) of sufficient quality for clarity? OK

Reviewer #2: only part of the results are reliable.

All figures lack sufficient definition.

**********

**Conclusions**

-Are the conclusions supported by the data presented?

-Are the limitations of analysis clearly described?

-Do the authors discuss how these data can be helpful to advance our understanding of the topic under study?

-Is public health relevance addressed?

Reviewer #1: -Are the conclusions supported by the data presented? OK

-Are the limitations of analysis clearly described? OK

-Do the authors discuss how these data can be helpful to advance our understanding of the topic under study? OK

-Is public health relevance addressed? OK

Reviewer #2: only approx half of the conclusions are well supported (those linked to TB-Profiler outputs).

**********

**Editorial and Data Presentation Modifications?**

Use this section for editorial suggestions as well as relatively minor modifications of existing data that would enhance clarity. If the only modifications needed are minor and/or editorial, you may wish to recommend “Minor Revision” or “Accept”.

Reviewer #1: (No Response)

Reviewer #2: (No Response)

**********

**Summary and General Comments**

Use this section to provide overall comments, discuss strengths/weaknesses of the study, novelty, significance, general execution and scholarship. You may also include additional comments for the author, including concerns about dual publication, research ethics, or publication ethics. If requesting major revision, please articulate the new experiments that are needed.

Reviewer #1: The authors have addressed each point in a clear and comprehensive manner. The revisions have significantly improved the clarity and overall quality of manuscript.

I have no further comments or concerns.

Reviewer #2: The revised version of the manuscript includes more raw data, as suggested during the first review. However, while specific attention was drawn to a pitfall in analyses, namely regarding the use of de novo assemblies for inferring SNPs presence, no change in methodology was proposed, and the clarification of the methodology and the raw data provided allows to think that several analyses (especially the phylogenetic analysis) are flawed. Added to this are some strange inconsistencies (mention of blastn for genome cleaning, and this only in the result section, use of SNP-sites to extract SNPs, improper reference to the supp table, absence of some supp information). In the end, provided information support only approximately half of the claims of the authors, and irrelevant information regarding methods, incorrect Suppl. Table numbering adds to the weaknesses of the study. Hence, even if the topic of the study is of interest, it seems dangerous to publish this study in its present form, due to the lack of data supporting clear reliability. If another version of the article were to be submitted, it should modify the whole genome analysis, or be restricted to the clear description of phenotypic vs genotypic data and to the outputs of TB-Profiler. Also, special attention should be put on consistency between methods mentioned in the results section and method having actually helped gather results.

Detailed comments:

-l.39 SNP-sites: this tool is not the one that can extract SNPs (it can only reduce the size of an alignment by extracting all positions for which all strains share the same SNP). In addition, also as detailed below, the whole applied procedure, namely reconstructing genomes by de novo assembly using Roary is suboptimal. As detailed in reviews such as Mehan et al, 2019, the authors should have used snippy or the --call_whole_genome argument of tbprofiler, or other all in one tools such as Phyresse or MTBseq

l.146-150 + l.160-161 : again, as mentioned in reviewer of version 1 of the article, de novo assemblies are suboptimal for extracting SNPs and should have been replaced by TBprofiler --call_whole_genome argument, or by snippy analysis. In addition, the authors then mention sequence alignment. This is strange because once SNPs have been extracted for all strains and combined in a single vcf file, there should be no need for such a step of sequences alignment: the extraction of SNPs from the genome creates artificial pseudo sequence of the same length, where only information at the same position may have a related origin. Destroying this alignment by an additional step can only introduce errors.

The authors argue that de novo assembly is necessary because L5 and L6 genomes include sequences that are absent from H37Rv. They have a point, but such assembly is only reliable when people have obtained long reads sequencing data. In addition, the highest number of additional genes that has been identified in L5 is 34 genes. This is little in comparison to the 4,173 genes consensually predicted. In contrast CheckM analysis focuses solely on 693 genes (see below).

l. 150 : improper syntax, and authors refer to coverage and not depth at this line -> to be changed.

l.157 : “SNP values” does not make sense. Replace by “SNP-distance values” or “pairwise-comparisons SNP numbers”

Statistical analyses and results l. 241 and following: there is no clear relevance of comparing the proportion f resistance isolates for different antibiotics.

l.182: genome cleaning using BLASTn makes no sense; in addition, it is not clear what “genome” refers to: assemblies or raw sequencing data? Furthermore, no such thing is described in the methods section.

l. 185: The S1 Table referred to at this line and the subsequent, as available in SUPPLEMENTARY MATERIALS.docx, is entitled “Supplementary Table S1: Comparison of the resistance rate of each antibiotic, with the average resistance rate of all other antibiotics tested.” It is Table S6 that contains CheckM outputs. But the information on the provided output is too scarce to be interpretable. Of note, CheckM focuses on highly conserved genes (apparently 693 genes, out of the more than 4,000 genes in M. tuberculosis H37Rv genome), and not at all on the totality of the genome, which means that Completeness in CheckM output does not relate to real genome completeness but rather to the extent to which highly conserved genes are actually present in the reconstructed genomes.

Subsequent descriptive statistics regarding SNP distance, supposedly available in Table S3, are only available in a very difficult to read format as labels of fig S1. Some values of these SNP distances are well above the expectations (up to 3029) which is highly suggestive of errors in the SNPs identification. Indeed others studies (for instance Guyeux et al, 2024) have detected a maximum interlineage distance of 2,100 SNPs while intra-L6 distance lied below 1,500 SNPs. Altogether, supporting information is still incomplete and some of the supporting information provided actually points out to substantial noise and errors in the analyses. Downstream analyses regarding pairwise distances (l.275 and following) are thus not reliable.

Linked to l. 263 and following: while authors claim that sublineage defining positions were manually checked, no related method is described, and the reference to Table S6 (actually most relevant data lie in Table S5) suggest that the only thing authors did is to look thoroughly at this table and not look at reads alignments. Still, the reference to 1 read out of 89 with a sublineage SNP suggests that they may have performed read alignment exploration, but no supporting data is provided. In another version of the article, the underlying method should be made clearly explicit.

l. 188: The expression “215.19% average sequencing coverage” does not make sense. If you use a percentage, it should be clear to what it is referring (i.e. percentage of what?)

l. 193 : lineage assignation: method (using TB-Profiler) should be reminded of.

l. 198 : “phylogenetic analysis”/Fig. S1. The figure should have a much better definition to be readable.

l. 217: Fig. 2: The figure still contains a blank part, as in version 1 of the article.

l.220 and following, it should be explained that the detailed analysis of Tb-profiler resistance data was only provided for 19 strains, or rather, this analysis should be extended to all samples. It is very unfortunate that for the 19 samples for which they are available, the phenotypic data are not put side by side with the genotypic resistance data. They should be.

l. L.329 “density of 951 SNPs” makes no sense. Rephrase.

Minor comments:

l.29 and l.62 Modify as in the introduction: Senegal is not the country of origin nore the birthplace of M. africanum, but it is indeed the country in which it was first described. It would be relevant in this context to sum up the results of Coscolla et al study (2021)

l. 48 confusing phrasing: not clear whether the streptomycin resistant strains is to be counted on the 16/19 isolates or not. It is also not clear whether strains carrying embC mutation Ala307Thr were identified as resistant phenotypically or not.

l. 51: as the data gathered offer a first detailed picture of M. afri diversity in Senegal, it is inappropriate to speak of an “evolving geographic structuration”. What is confirmed is the predominance of L6, and some diversity among L6 strains. Or if the authors want to argue on the evolution of the L presence in Senegal, they should more carefully report the dates of the different references and the trends revealed by their confrontation.

The discrepancy between DST and WGS is not necessarily a sign that phenotypic DST is not effective. It could be a sign of difficulty to obtain WGS data actually corresponding to clinical samples due to the multiple steps of data transmission and a suboptimal connexion between samples and labels. The conclusion is thus an overstatement.

l. 63 : Correct “the majority of tuberculosis cases” for a significant part of tuberculosis cases, as it was only shown for Guinea-Bissau that M. africanum contributed to more than 40% of TB cases.

l.186 spolygotyping -> spoligotyping

**********

PLOS authors have the option to publish the peer review history of their article (what does this mean?). If published, this will include your full peer review and any attached files.

If you choose “no”, your identity will remain anonymous but your review may still be made public.

**Do you want your identity to be public for this peer review?**  For information about this choice, including consent withdrawal, please see our Privacy Policy.

Reviewer #1: No

Reviewer #2: No

**Figure resubmission:**

While revising your submission, we strongly recommend that you use PLOS’s NAAS tool (https://ngplosjournals.pagemajik.ai/artanalysis) to test your figure files. NAAS can convert your figure files to the TIFF file type and meet basic requirements (such as print size, resolution), or provide you with a report on issues that do not meet our requirements and that NAAS cannot fix.

After uploading your figures to PLOS’s NAAS tool - https://ngplosjournals.pagemajik.ai/artanalysis, NAAS will process the files provided and display the results in the "Uploaded Files" section of the page as the processing is complete. If the uploaded figures meet our requirements (or NAAS is able to fix the files to meet our requirements), the figure will be marked as "fixed" above. If NAAS is unable to fix the files, a red "failed" label will appear above. When NAAS has confirmed that the figure files meet our requirements, please download the file via the download option, and include these NAAS processed figure files when submitting your revised manuscript.

**Reproducibility:**

To enhance the reproducibility of your results, we recommend that authors of applicable studies deposit laboratory protocols in protocols.io, where a protocol can be assigned its own identifier (DOI) such that it can be cited independently in the future. Additionally, PLOS ONE offers an option to publish peer-reviewed clinical study protocols. Read more information on sharing protocols at https://plos.org/protocols?utm_medium=editorial-email&utm_source=authorletters&utm_campaign=protocols

# Author comment: Author response to Decision Letter 2

**DOI:** 10.1371/journal.pntd.0014770.r004

9 Apr 2026

Attachment Submitted filename: Response to Reviewers.docx

# Peer review: Decision Letter 2

**DOI:** 10.1371/journal.pntd.0014770.r005

**Authors:** Mathieu Picardeau

1 May 2026

PNTD-D-25-01817R2

Genomic microdiversity of Mycobacterium africanum in Sénégal.

PLOS Neglected Tropical Diseases

Dear Dr. Drancourt,

Thank you for submitting your manuscript to PLOS Neglected Tropical Diseases. After careful consideration, we feel that it has merit but does not fully meet PLOS Neglected Tropical Diseases's publication criteria as it currently stands. Therefore, we invite you to submit a revised version of the manuscript that addresses the points raised during the review process.

Please submit your revised manuscript within by May 31 2026 11:59PM. If you will need more time than this to complete your revisions, please reply to this message or contact the journal office at plosntds@plos.org. When you're ready to submit your revision, log on to https://www.editorialmanager.com/pntd/ and select the 'Submissions Needing Revision' folder to locate your manuscript file.

Please include the following items when submitting your revised manuscript:

* A letter that responds to each point raised by the editor and reviewer(s). You should upload this letter as a separate file labeled 'Response to Reviewers'. This file does not need to include responses to any formatting updates and technical items listed in the 'Journal Requirements' section below.

* A marked-up copy of your manuscript that highlights changes made to the original version. You should upload this as a separate file labeled 'Revised Manuscript with Track Changes'.

* An unmarked version of your revised paper without tracked changes. You should upload this as a separate file labeled 'Manuscript'.

If you would like to make changes to your financial disclosure, competing interests statement, or data availability statement, please make these updates within the submission form at the time of resubmission. Guidelines for resubmitting your figure files are available below the reviewer comments at the end of this letter.

We look forward to receiving your revised manuscript.

Kind regards,

Mathieu Picardeau

Section Editor

PLOS Neglected Tropical Diseases

Shaden Kamhawi

co-Editor-in-Chief

PLOS Neglected Tropical Diseases

orcid.org/0000-0003-4304-636XX

Paul Brindley

co-Editor-in-Chief

PLOS Neglected Tropical Diseases

orcid.org/0000-0003-1765-0002

**Reviewers' Comments:**

Reviewer's Responses to Questions

**Key Review Criteria Required for Acceptance?**

As you describe the new analyses required for acceptance, please consider the following:

**Methods**

-Are the objectives of the study clearly articulated with a clear testable hypothesis stated?

-Is the study design appropriate to address the stated objectives?

-Is the population clearly described and appropriate for the hypothesis being tested?

-Is the sample size sufficient to ensure adequate power to address the hypothesis being tested?

-Were correct statistical analysis used to support conclusions?

-Are there concerns about ethical or regulatory requirements being met?

Reviewer #2: Methods used to describe diversity still rely on strange proxies

l.299: median minus 2 std dev used extensively as a proxy for diversity... but:

*std dev is defined according to the mean, so if combined to Something, this should be the mean.

*if one wants to capture the global trend of a sample for a quantitative parameter, mean is chosen when this parameter is normally distribued, and median is preferred otherwise. Mean minus 2 std dev identifies the threshold for 2,5% of strains with the lowest diversity in the subsample, and does not capture any global trend

*a standard value used for diversity assessment is the presence of strains with less than 12 SNPs distance, suggestive of relatively recent transmission, in accordance with standards where recent transmission was independently supported (Walker et al, 2013, for instance). There is no mention of this standard here

Methods regarding resistance prediction suffer from flaws: Many mutations described as responsible for resistance in the text and tables are in fact annotated as "Uncertain significance" in the WHO catalog. Especially those present in the supposedly MDR strain, and those linked to suppsed ethambutol resistance, that contradict phenotypic results. In fact, as such, these should be considered as not conferring resistance. Please revisit all resistance annotations in light with WHO catalog of such resistance mutations, and annotate in light of their significance or withdraw all mutations that are not clearly associated with resistance.

Methods regarding genome inferences are still partly flawed: the authors should be aware that the genomes they reconstructed through their bioinformatical analyses are faked, copying H37Rv genome and replacing only the ny where SNPs or indels have been identified. This type of faked genome is highly useful for phylogeny, but should not be used and presented as real : no exploration has been made of the number and position of IS6110, deletion regions, etc. Hence the genome sizes reported in S1 table should be withdrawn. Accordingly, there is no relevance to present dDDH data on such genomes. This information should also be withdrawn for result section.

-it is not clear whether SNPs used for phylogeny etc include PE-PPE or not, and other régions possibly associated with imperfect read alignment (see again Meehan 2019). Please describe more precisely.

**********

**Results**

-Does the analysis presented match the analysis plan?

-Are the results clearly and completely presented?

-Are the figures (Tables, Images) of sufficient quality for clarity?

Reviewer #2: some data do not match : SN01 coverage indicated to be 41 in S1 table, and 21 in S4 table. All compiled data should be re-checked.

problems related to methodology as mentionned above.

the long presentation of % of resistance with so small sample sizes is still misleading and of relatively low benefit. And even more misleading considering that most are not resistant at all. This part could be reduced to the mention of mutations observed in several samples, and their potential impact.

**********

**Conclusions**

-Are the conclusions supported by the data presented?

-Are the limitations of analysis clearly described?

-Do the authors discuss how these data can be helpful to advance our understanding of the topic under study?

-Is public health relevance addressed?

Reviewer #2: Conclusions regarding diversity are well supported.

Conclusions regarding resistance should be re-examined.

**********

**Editorial and Data Presentation Modifications?**

Use this section for editorial suggestions as well as relatively minor modifications of existing data that would enhance clarity. If the only modifications needed are minor and/or editorial, you may wish to recommend “Minor Revision” or “Accept”.

Reviewer #2: Some English writing such as

l. 344 : small sample size (not "effective")

**********

**Summary and General Comments**

Use this section to provide overall comments, discuss strengths/weaknesses of the study, novelty, significance, general execution and scholarship. You may also include additional comments for the author, including concerns about dual publication, research ethics, or publication ethics. If requesting major revision, please articulate the new experiments that are needed.

Reviewer #2: The article by Drancourt rev.2 et al has been efficiently purged from major biases included in previous versions. It now presents a phylogeny of Mycobacterium africanum isolates identified in Senegal that seems reliable. The description of their geographic distribution is backed up by a clear map presenting both population sizes and diversity. Information on coverage, reads, is now much clearer and precise.

The disappearance of major defects revealed some other major points that should imperatively be adressed before publication:

Major comments:

-Results describing diversity still rely on strange proxies

l.299: median minus 2 std dev used extensively as a proxy for diversity... but:

*std dev is defined according to the mean, so if combined to Something, this should be the mean.

*if one wants to capture the global trend of a sample for a quantitative parameter, mean is chosen when this parameter is normally distribued, and median is preferred otherwise. Mean minus 2 std dev identifies the threshold for 2,5% of strains with the lowest diversity in the subsample, and does not capture any global trend

*a standard value used for diversity assessment is the presence of strains with less than 12 SNPs distance, suggestive of relatively recent transmission, in accordance with standards where recent transmission was independently supported (Walker et al, 2013, for instance). There is no mention of this standard here

Hence the results on diversity need to be revised.

-some data do not match : SN01 coverage indicated to be 41 in S1 table, and 21 in S4 table. All compiled data should be re-checked.

-Many mutations described as responsible for resistance in the text and tables are in fact annotated as "Uncertain significance" in the WHO catalog. Especially those present in the supposedly MDR strain, and those linked to suppsed ethambutol resistance, that contradict phenotypic results. In fact, as such, these should be considered as not conferring resistance. Please revisit all resistance annotations in light with WHO catalog of such resistance mutations, and annotate in light of their significance or withdraw all mutations that are not clearly associated with resistance. This should reshape discussion.

-the authors should be aware that the genomes they reconstructed through their bioinformatical analyses are faked, copying H37Rv genome and replacing only the ny where SNPs or indels have been identified. This type of faked genome is highly useful for phylogeny, but should not be used and presented as real : no exploration has been made of the number and position of IS6110, deletion regions, etc. Hence the genome sizes reported in S1 table should be withdrawn.Accordingly, there is no relevance to present dDDH data on such genomes. This information should also be withdrawn for result section.

Minor points:

-the long presentation of % of resistance with so small sample sizes is still misleading and of relatively low benefit. And even more misleading considering that most are not resistant at all. This part could be reduced to the mention of mutations observed in several samples, and their potential impact.

-l. 344 : small sample size (not "effective")

-it is not clear whether SNPs used for phylogeny etc include PE-PPE or not, and other régions possibly associated with imperfect read alignment (see again Meehan 2019). Please describe more precisely.

-mutation frequency absent from table S1

-the high contamination and low read number supporting resistance in SN48 should be more clearly mentionned in the text.

**********

PLOS authors have the option to publish the peer review history of their article (what does this mean?). If published, this will include your full peer review and any attached files.

If you choose “no”, your identity will remain anonymous but your review may still be made public.

**Do you want your identity to be public for this peer review?**  For information about this choice, including consent withdrawal, please see our Privacy Policy.

Reviewer #2: No

[NOTE: If reviewer comments were submitted as an attachment file, they will be attached to this email and accessible via the submission site. Please log into your account, locate the manuscript record, and check for the action link "View Attachments". If this link does not appear, there are no attachment files.]

**Figure resubmission:**  While revising your submission, we strongly recommend that you use PLOS’s NAAS tool (https://ngplosjournals.pagemajik.ai/artanalysis) to test your figure files. NAAS can convert your figure files to the TIFF file type and meet basic requirements (such as print size, resolution), or provide you with a report on issues that do not meet our requirements and that NAAS cannot fix.

After uploading your figures to PLOS’s NAAS tool - https://ngplosjournals.pagemajik.ai/artanalysis, NAAS will process the files provided and display the results in the "Uploaded Files" section of the page as the processing is complete. If the uploaded figures meet our requirements (or NAAS is able to fix the files to meet our requirements), the figure will be marked as "fixed" above. If NAAS is unable to fix the files, a red "failed" label will appear above. When NAAS has confirmed that the figure files meet our requirements, please download the file via the download option, and include these NAAS processed figure files when submitting your revised manuscript.

**Reproducibility:**

To enhance the reproducibility of your results, we recommend that authors of applicable studies deposit laboratory protocols in protocols.io, where a protocol can be assigned its own identifier (DOI) such that it can be cited independently in the future. Additionally, PLOS ONE offers an option to publish peer-reviewed clinical study protocols. Read more information on sharing protocols at https://plos.org/protocols?utm_medium=editorial-email&utm_source=authorletters&utm_campaign=protocols

# Author comment: Author response to Decision Letter 3

**DOI:** 10.1371/journal.pntd.0014770.r006

30 Jun 2026

Attachment Submitted filename: R3 rebutal letter.docx

# Editor report: Decision Letter 3

**DOI:** 10.1371/journal.pntd.0014770.r007

**Authors:** Mathieu Picardeau

25 Aug 2026

PNTD-D-25-01817R3Genomic microdiversity of Mycobacterium africanum in Sénégal.PLOS Neglected Tropical Diseases Dear Dr. Drancourt, Thank you for submitting your manuscript to PLOS Neglected Tropical Diseases. After careful consideration, we feel that it has merit but does not fully meet PLOS Neglected Tropical Diseases's publication criteria as it currently stands. Therefore, we invite you to submit a revised version of the manuscript that addresses the points raised during the review process. Please submit your revised manuscript by Sep 24 2026 11:59PM. If you will need more time than this to complete your revisions, please reply to this message or contact the journal office at plosntds@plos.org. When you're ready to submit your revision, log on to https://www.editorialmanager.com/pntd/ and select the 'Submissions Needing Revision' folder to locate your manuscript file. Please include the following items when submitting your revised manuscript:* A letter that responds to each point raised by the editor and reviewer(s). You should upload this letter as a separate file labeled 'Response to Reviewers'. This file does not need to include responses to any formatting updates and technical items listed in the 'Journal Requirements' section below.* A marked-up copy of your manuscript that highlights changes made to the original version. You should upload this as a separate file labeled 'Revised Manuscript with Track Changes'.* An unmarked version of your revised paper without tracked changes. You should upload this as a separate file labeled 'Manuscript'. If you would like to make changes to your financial disclosure, competing interests statement, or data availability statement, please make these updates within the submission form at the time of resubmission. Guidelines for resubmitting your figure files are available below the reviewer comments at the end of this letter. As the corresponding author, your ORCID iD is verified in the submission system and will appear in the published article. PLOS supports the use of ORCID, and we encourage all coauthors to register for an ORCID iD and use it as well. Please encourage your coauthors to verify their ORCID iD within the submission system before final acceptance, as unverified ORCID iDs will not appear in the published article. Only the individual author can complete the verification step; PLOS staff cannot verify ORCID iDs on behalf of authors.We look forward to receiving your revised manuscript. Kind regards,  Mathieu PicardeauSection EditorPLOS Neglected Tropical Diseases

Shaden Kamhawi

co-Editor-in-Chief

PLOS Neglected Tropical Diseases

orcid.org/0000-0003-4304-636XX

Paul Brindley

co-Editor-in-Chief

PLOS Neglected Tropical Diseases

orcid.org/0000-0003-1765-0002

**Reviewers' comments:**

comments from reviewer 3:

The authors took in consideration previous remarks made by reviewers, the paper is now suitable for publication.

lane 71: the fact that L5 is present in Gambia and rarely in Senegal (Lane 71-72) is not evident to the reviewer. Please add reference

lane 107: reference for the CTAB method is missing, please add reference.

lane 229: one space is missing in "DSTin 16". it should be : "DST in 16"

As far as I'm concerned, the main conclusion of this study is that L5 is virtually absent from Senegal, whereas it is present (according to the authors) in The neighboring Gambia, which seems—at least at first glance—to share quite a few similarities with Senegal. This could be discussed in a conclusive sentence.

[NOTE: If reviewer comments were submitted as an attachment file, they will be attached to this email and accessible via the submission site. Please log into your account, locate the manuscript record, and check for the action link "View Attachments". If this link does not appear, there are no attachment files.] **Figure resubmission:**  While revising your submission, we strongly recommend that you use PLOS’s NAAS tool (https://ngplosjournals.pagemajik.ai/artanalysis) to test your figure files. NAAS can convert your figure files to the TIFF file type and meet basic requirements (such as print size, resolution), or provide you with a report on issues that do not meet our requirements and that NAAS cannot fix.

After uploading your figures to PLOS’s NAAS tool - https://ngplosjournals.pagemajik.ai/artanalysis, NAAS will process the files provided and display the results in the "Uploaded Files" section of the page as the processing is complete. If the uploaded figures meet our requirements (or NAAS is able to fix the files to meet our requirements), the figure will be marked as "fixed" above. If NAAS is unable to fix the files, a red "failed" label will appear above. When NAAS has confirmed that the figure files meet our requirements, please download the file via the download option, and include these NAAS processed figure files when submitting your revised manuscript. **Reproducibility:**  To enhance the reproducibility of your results, we recommend that authors of applicable studies deposit laboratory protocols in protocols.io, where a protocol can be assigned its own identifier (DOI) such that it can be cited independently in the future. Additionally, PLOS ONE offers an option to publish peer-reviewed clinical study protocols. Read more information on sharing protocols at https://plos.org/protocols?utm_medium=editorial-email&utm_source=authorletters&utm_campaign=protocols

# Author comment: Author response to Decision Letter 4

**DOI:** 10.1371/journal.pntd.0014770.r008

17 Sep 2026

Attachment Submitted filename: R4 rebutal letter.docx

# Editor report: Decision Letter 4

**DOI:** 10.1371/journal.pntd.0014770.r009

**Authors:** Mathieu Picardeau

20 Sep 2026

Dear Prof Drancourt,

We are pleased to inform you that your manuscript 'Genomic microdiversity of Mycobacterium africanum in Sénégal.' has been provisionally accepted for publication in PLOS Neglected Tropical Diseases.

Before your manuscript can be formally accepted you will need to complete some formatting changes, which you will receive in a follow up email. A member of our team will be in touch with a set of requests.

Please note that your manuscript will not be scheduled for publication until you have made the required changes, so a swift response is appreciated.

IMPORTANT: The editorial review process is now complete. PLOS will only permit corrections to spelling, formatting or significant scientific errors from this point onwards. Requests for major changes, or any which affect the scientific understanding of your work, will cause delays to the publication date of your manuscript.

Should you, your institution's press office or the journal office choose to press release your paper, you will automatically be opted out of early publication. We ask that you notify us now if you or your institution is planning to press release the article. All press must be co-ordinated with PLOS.

Thank you again for supporting Open Access publishing; we are looking forward to publishing your work in PLOS Neglected Tropical Diseases.

Best regards,

Mathieu Picardeau

Section Editor

PLOS Neglected Tropical Diseases

Shaden Kamhawi

co-Editor-in-Chief

PLOS Neglected Tropical Diseases

orcid.org/0000-0003-4304-636XX

Paul Brindley

co-Editor-in-Chief

PLOS Neglected Tropical Diseases

orcid.org/0000-0003-1765-0002

***********************************************************

# Editor report: Acceptance letter

**DOI:** 10.1371/journal.pntd.0014770.r010

**Authors:** Mathieu Picardeau

Dear Prof Drancourt,

We are delighted to inform you that your manuscript, "Genomic microdiversity of Mycobacterium africanum in Sénégal.," has been formally accepted for publication in PLOS Neglected Tropical Diseases.

We have now passed your article onto the PLOS Production Department who will complete the rest of the publication process. All authors will receive a confirmation email upon publication.

The corresponding author will soon be receiving a typeset proof for review, to ensure errors have not been introduced during production. Please review the PDF proof of your manuscript carefully, as this is the last chance to correct any scientific or type-setting errors. Please note that major changes, or those which affect the scientific understanding of the work, will likely cause delays to the publication date of your manuscript. Note: Proofs for Front Matter articles (Editorial, Viewpoint, Symposium, Review, etc...) are generated on a different schedule and may not be made available as quickly.

Soon after your final files are uploaded, the early version of your manuscript will be published online unless you opted out of this process. The date of the early version will be your article's publication date. The final article will be published to the same URL, and all versions of the paper will be accessible to readers.

For Research Articles, you will receive an invoice from PLOS for your publication fee after your manuscript has reached the completed accept phase. If you receive an email requesting payment before acceptance or for any other service, this may be a phishing scheme. Learn how to identify phishing emails and protect your accounts at https://explore.plos.org/phishing.

Thank you again for supporting open-access publishing; we are looking forward to publishing your work in PLOS Neglected Tropical Diseases.

Best regards,

Shaden Kamhawi

co-Editor-in-Chief

PLOS Neglected Tropical Diseases

Paul Brindley

co-Editor-in-Chief

PLOS Neglected Tropical Diseases
